# A Comprehensive Study of the Key Enumeration Problem

**DOI:** 10.3390/e21100972

**Published:** 2019-10-05

**Authors:** Ricardo Villanueva-Polanco

**Affiliations:** Computer Science Department, Universidad del Norte, Barranquilla 080001, Colombia; rpolanco@uninorte.edu.co

**Keywords:** cold boot attacks, key recovery, enumeration, algorithms

## Abstract

In this paper, we will study the key enumeration problem, which is connected to the key recovery problem posed in the cold boot attack setting. In this setting, an attacker with physical access to a computer may obtain noisy data of a cryptographic secret key of a cryptographic scheme from main memory via this data remanence attack. Therefore, the attacker would need a key-recovery algorithm to reconstruct the secret key from its noisy version. We will first describe this attack setting and then pose the problem of key recovery in a general way and establish a connection between the key recovery problem and the key enumeration problem. The latter problem has already been studied in the side-channel attack literature, where, for example, the attacker might procure scoring information for each byte of an Advanced Encryption Standard (AES) key from a side-channel attack and then want to efficiently enumerate and test a large number of complete 16-byte candidates until the correct key is found. After establishing such a connection between the key recovery problem and the key enumeration problem, we will present a comprehensive review of the most outstanding key enumeration algorithms to tackle the latter problem, for example, an optimal key enumeration algorithm (OKEA) and several nonoptimal key enumeration algorithms. Also, we will propose variants to some of them and make a comparison of them, highlighting their strengths and weaknesses.

## 1. Introduction

A side-channel attack may be defined as any attack by which an attacker is able to obtain private information of a cryptographic algorithm from its implementation instead of exploiting weaknesses in the implemented algorithm itself. Most of these attacks are based on a divide-and-conquer approach through which the attacker obtains ranking information about the chunks of the secret key and then uses such information to construct key candidates for that key. This secret key is the result of the concatenation of all the key parts, while a chunk candidate is a possible value of a key part that is chosen because the attack suggests a good probability for that value to be correct. Particularly, we will focus on a particular side-channel attack, known as cold boot attack. This is a data remanence attack in which the attacker is able to read sensitive data from a source of computer memory after supposedly having been deleted. More specifically, exploiting the data remanence property of dynamic random-access memories (DRAMs), an attacker with physical access to a computer, may procure noisy data of a secret key from main memory via this attack vector. Hence, after obtaining such data, the attacker’s main task is to recover the secret key from its noisy version. As it will be revealed by the literature in Section 2, the research effort, after the initial work showing the practicability of cold boot attacks [1], has focused on designing tailor-made algorithms for efficiently recovering keys from noisy versions for a range of different cryptographic schemes whilst exploring the limits of how much noise can be tolerated.

The above discussion raises the following question: can we devise a general approach to key recovery in the cold boot attack setting, i.e., a general algorithmic strategy that can be applied to recovering keys from noisy versions of those keys for a range of different cryptographic schemes? In this research paper, we work toward answering this question by studying the key enumeration problem, which is connected to the key recovery problem in the cold boot attack setting. Therefore, this paper, to the best of our knowledge, is the first to present a comprehensive review of the most outstanding key enumeration algorithms to tackle the key enumeration problem. Explicitly, our major contributions in this research work are the following:

1. We present the key recovery problem in a general way and establish a connection between the key recovery problem and the key enumeration problem.
2. We describe the most outstanding key enumeration algorithms methodically and in detail and also propose variants to some of them. The algorithms included in this study are an optimal key enumeration algorithm (OKEA); a bounded-space near-optimal key enumeration algorithm; a simple stack-based, depth-first key enumeration algorithm; a score-based key enumeration algorithm; a key enumeration algorithm using histograms; and a quantum key enumeration algorithm. For each studied algorithm, we describe its inner functioning, showing its functional and qualitative features, such as memory consumption, amenability to parallelization; and scalability.
3. Finally, we make an experimental comparison of all the implemented algorithms, drawing special attention to their strengths and weaknesses. In our comparison, we benchmark all the implemented algorithms by running them in a common scenario to measure their overall performance.

Note that the goal of this research work is not only to study the key enumeration problem and its connection to the key recovery problem but also to show the gradual development of designing key enumeration algorithms, i.e., our review also focuses on pointing out the most important design principles to look at when designing key enumeration algorithms. Therefore, our review examines the most outstanding key enumeration algorithms methodically, via describing their inner functioning, the algorithm-related data structures, and the benefits and drawbacks from using such data structures. Particularly, this careful examination shows us that, by properly using data structures and by making the restriction on the order in which the key candidates are enumerated less strict, we may devise better key enumeration algorithms in terms of overall performance, scalability, and memory consumption. This observation is substantiated in our experimental comparison.

This paper is organised as follows. In Section 2, we will first describe the cold boot attack setting and the attack model we will use throughout this paper. In Section 3,we will describe the key recovery problem in a general way and establish a connection between the key recovery problem and the key enumeration problem. In Section 4, we will examine several key enumeration algorithms to tackle the key enumeration problem methodically and in detail, e.g., an optimal key enumeration algorithm (OKEA), a bounded-space near-optimal key enumeration algorithm, a quantum key enumeration algorithm, and  variants of other key enumeration algorithms. In Section 5, we will make a comparison of them, highlighting their strengths and weaknesses. Finally, in Section 6, we will draw some conclusions and give some future research lines.

## 2. Cold Boot Attacks

A cold boot attack is a type of data remanence attack by which sensitive data are read from a computer’s main memory after supposedly having been deleted. This attack relies on the data remanence property of DRAMs that allows an attacker to retrieve memory contents that remain readable in the seconds to minutes after power has been removed. Since this attack was first described in the literature by Halderman et al. nearly a decade ago [1], it has received significant attention. In this setting, more specifically, an attacker with physical access to a computer can retrieve content from a running operating system after performing a cold reboot to restart the machine, i.e., not shutting down the operating system in an orderly manner. Since the operating system was shut down improperly, it will skips file system synchronization and other activities that would occur on an orderly shutdown. Therefore, following a cold reboot, such an attacker may use a removable disk to boot a lightweight operating system and then copy stored data in memory to a file. As another option or possibility, such an attacker may take the memory modules off the original computer and quickly put them in a compatible computer under the attacker’s control, which is then started and put into a state of readiness for operation in order to access the memory content. Also, this attacker may perform a further analysis against the data that was dumped from memory to find various sensitive information, such as cryptographic keys contained in it [1]. This task may be performed by making use of various forms of key finding algorithms [1]. Unfortunately for such an attacker, the bits in memory will degrade once the computer’s power is interrupted. Therefore, if the adversary retrieves any data from the computer’s main memory after the power is cut off, the extracted data will probably have random bit variations. This is, the data will be noisy, i.e., differing from the original data.

The lapse of time for which cell memory values are maintained while the machine is off depends on the particular memory type and the ambient temperature. In fact, the research paper [1] reported the results of multiple experiments that show that, at normal operating temperatures (25.5 °C to 44.1 °C), there is little corruption within the first few seconds but this phase is then followed by a quick decay. Nevertheless, by employing cooling techniques on the memory chips, the period of mild corruption can be extended. For instance, by spraying compressed air onto the memory chips, they achieved an experiment at 50 °C and showed that less than 0.1% of bits degrade within the first minute. At temperatures of approximately −196 °C, attained by the use of liquid nitrogen, less than 0.17% of bits decay within the first hour. Remarkably, once power is switched off, the memory will be divided into regions and each region will have a “ground state”, which is associated with a bit. In a 0 ground state, the 1 bits will eventually decay to 0 bits while the probability of a 0 bit switching to a 1 bit is very small but not vanishing (a common probability is circa 0.001 [1]). When the ground state is 1, the opposite is true.

From the above discussion, it follows that only a noisy version of the original key may be retrievable from main memory once the attacker discovers the location of the data in it, so the main task of the attacker then is to tackle the mathematical problem of recovering the original key from a noisy version of that key. Therefore, the centre of interest of the research community after the initial work pointing out the feasibility of cold boot attacks [1] has been to develop bespoke algorithms for efficiently recovering keys from noisy versions of those keys for a range of different cryptographic schemes whilst exploring the limits of how much noise can be tolerated.

Heninger and Shacham [2] focused on the case of RSA keys, introducing an efficient algorithm based on Hensel lifting to exploit redundancy in the typical RSA private key format. This work was followed up by Henecka, May, and Meurer [3] and by Paterson, Polychroniadou, and Sibborn [4], with both research papers also paying particular attention to the mathematically highly structured RSA setting. The latter research paper, in particular, indicated the asymmetric nature of the error channel intrinsic to the cold boot setting and presented the problem of key recovery for cold boot attacks in an information theoretic manner.

On the other hand, Lee et al. [5] were the first to discuss cold boot attacks in the discrete logarithm setting. They assumed that an attacker had access to the public key $g^{x}$, a noisy version of the private key x, and that such an attacker knew an upper bound for the number of errors in the private key. Since the latter assumption might not be realistic and the attacker did not have access to further redundancy, their proposed algorithm would likely be unable to recover keys in the true cold boot scenario, i.e., only assuming a bit-flipping model. This work was followed up by Poettering and Sibborn [6]. They exploited redundancies present in the in-memory private key encodings from two elliptic curve cryptography (ECC) implementations from two Transport Layer Security (TLS) libraries, OpenSSL and PolarSSL, and introduced cold boot key-recovery algorithms that were applicable to the true cold boot scenario.

Other research papers have explored cold boot attacks in the symmetric key setting, including Albrecht and Cid [7], who centred on the recovery of symmetric encryption keys in the cold boot setting by employing polynomial system solvers, and Kamal and Youssef [8], who applied SAT solvers to the same problem.

Finally, recent research papers have explored cold boot attacks on post-quantum cryptographic schemes. The paper by Albrecht et al. [9] evaluated schemes based on the ring—and module—variants of the Learning with Errors (LWE) problem. In particular, they looked at two cryptographic schemes: the Kyber key encapsulation mechanism (KEM) and New Hope KEM. Their analysis focused on two encodings to store LWE keys. The first encoding stores polynomials in coefficient form directly in memory, while the second encoding performs a number theoretic transform (NTT) on the key before storing it. They showed that, at a 1% bit-flip rate, a cold boot attack on Kyber KEM parameters had a cost of $2^{43}$ operations when the second encoding is used for key storage compared to $2^{70}$ operations with the first encoding. On the other hand, the paper by Paterson et al. [10] focused on cold boot attacks on NTRU. Particularly the authors of the research paper [10] were the first that used a combination of key enumeration algorithms to tackle the key recovery problem. Their cold boot key-recovery algorithms were applicable to the true cold boot scenario and exploited redundancies found in the in-memory private key representations from two popular NTRU implementations. This work was followed up by that of Villanueva-Polanco [11], which studied cold boot attacks against the strongSwan implementation of the BLISS signature scheme and presented key-recovery algorithms based on key enumeration algorithms for the in-memory private key encoding used in this implementation.

### Cold Boot Attack Model

Our cold boot attack model assumes that the adversary can procure a noisy version of the encoding of a secret key used to store it in memory. We further assume that the corresponding public parameters are known exactly, without noise. We do not take into consideration here the significant problem of how to discover the exact place or position of the appropriate region of memory in which the secret key bits are stored, though this would be a consideration of great significance in practical attacks. Our goal is then to recover the secret key. Note that it is sufficient to obtain a list of key candidates in which the true secret key is located, since we can always test a candidate by executing known algorithms linked to the scheme we are attacking.

We assume throughout that a 0 bit of the original secret key will flip to a 1 with probability α=P(0→1) and that a 1 bit of the original private key will flip with probability β=P(1→0). We do not assume that α=β; indeed, in practice, one of these values may be very small (e.g., 0.001) and relatively stable over time while the other increases over time. Furthermore, we assume that the attacker knows the values of α and β and that they are fixed across the region of memory in which the private key is located. These assumptions are reasonable in practice: one can estimate the error probabilities by looking at a region where the memory stores known values, for example, where the public key is located, and where the regions are typically large.

## 3. Key Recovery Problem

### 3.1. Some Definitions

We define an array *A* as a data structure consisting of a finite sequence of values of a specified type, i.e., $A=[a_{0},\ldots ,a_{n_{A}-1}]$. The length of an array, $n_{A}$, is established when the array is created. After creation, its length is fixed. Each item in an array is called an element, and each element is accessed by its numerical index, i.e., $A\left[i\right]=a_{i}$, with $0\leq i<n_{A}$. Let $A^{0}=\left[a_{0}^{0},\ldots ,a_{n_{0}-1}^{0}\right]$ and $A_{1}=\left[a_{0}^{1},\ldots ,a_{n_{1}-1}^{1}\right]$ be two arrays of elements of a specified type. The associative operation ‖ is defined as follows.

$$\left[a_{0}^{0},\ldots ,a_{n_{0}-1}^{0}\right]\|\left[a_{0}^{1},\ldots ,a_{n_{1}-1}^{1}\right]=\left[a_{0}^{0},\ldots ,a_{n_{0}-1}^{0},a_{0}^{1},\ldots ,a_{n_{1}-1}^{1}\right].$$

Both a list *L* and a table T are defined as a resizable array of elements of a specified type. Given a list $L=[e_{0},\ldots ,e_{n_{l}-1}]$, this data structure supports the following methods.

- The method L.size() returns the number of elements in this list, i.e., the value $n_{l}$.
- The method $L.\mathrm{add}(e_{n_{l}})$ appends the specified element $e_{n_{l}}$ to the end of this list, i.e., $L=[e_{0},e_{1},\ldots ,e_{n_{l}}]$ after this method returns.
- The method L.get(j), with 0≤j<L.size(), returns the element at the specified position *j* in this list, i.e., $e_{j}.$
- The method L.clear() removes all the elements from this list. The list will be empty after this method returns, i.e., L=[].

### 3.2. Problem Statement

Let us suppose that a noisy version of the encoding of the secret key $r=b_{0}b_{1}b_{2}\ldots b_{W}$ can be represented as a concatenation of N=W/w chunks, each on *w* bits. Let us name the chunks $r^{0},r^{1},\ldots ,r^{N-1}$ so that $r^{i}=b_{i\cdot w}b_{i\cdot w+1}\ldots b_{i\cdot w+(w-1)}$. Additionally, we suppose there is a key-recovery algorithm that constructs key candidates c for the encoding of the secret key and that these key candidates c can also be represented by concatenations of chunks $c^{0},c^{1},\ldots ,c^{N-1}$ in the same way.

The method of maximum likelihood (ML) estimation then suggests picking as c the value that maximizes P(c|r). Using Bayes’ theorem, this can be rewritten as $P\left(c|r\right)=\frac{P(r|c)P(c)}{P(r)}.$ Note that P(r) is a constant and that P(c) is also a constant, independent of c. Therefore, the ML estimation suggests picking as c the value that maximizes $P\left(r|c\right)={\left(1-\alpha \right)}^{n_{00}}\alpha^{n_{01}}\beta^{n_{10}}{\left(1-\beta \right)}^{n_{11}},$ where $n_{00}$ denotes the number of positions where both c and r contain a 0 bit and where $n_{01}$ denotes the number of positions where c contains a 0 bit and r contains a 1 bit, etc. Equivalently, we may maximize the log of these probabilities, viz. $\log\left(P\left(r|c\right)\right)=n_{00}\log\left(1-\alpha \right)+n_{01}\log\alpha +n_{10}\log\beta +n_{11}\log\left(1-\beta \right).$ Therefore, given a candidate c, we can assign it a score, namely $S_{r}\left(c\right):=\log\left(P\left(r|c\right)\right)$.

Assuming that each of the, at most, $2^{w}$ candidate values for chunk $c^{i}$, 0≤i<N, can be enumerated, then its own score also can be calculated as $S_{r^{i}}^{\prime }\left(c^{i}\right)=n_{00}^{i}\log\left(1-\alpha \right)+n_{01}^{i}\log\alpha +n_{10}^{i}\log\beta +n_{11}^{i}\log\left(1-\beta \right),$ where the $n_{ab}^{i}$ values count occurrences of bits across the $i_{th}$ chunks $c^{i}$ and $r^{i}$. Therefore, we have $S_{r}\left(c\right)=\sum_{i=0}^{N-1}S_{r^{i}}^{\prime }\left(c^{i}\right)$. Hence, we may assume we have access to N lists of chunk candidates, where each list contains up to $2^{w}$ entries. A chunk candidate is defined as a 2-tuple of the form (score,value), where the first component score is a real number (candidate score) while the second component value is an array of *w*-bit strings (candidate value). The question then becomes can we design efficient algorithms that traverse the lists of chunk candidates to combine chunk candidates $c^{i}$, obtaining complete key candidates c having high total scores obtained by summation? This question has been previously addressed in the side-channel analysis literature, with a variety of different algorithms being possible to solve this problem and the related problem known as key rank estimation [12,13,14,15,16,17,18,19,20,21,22,23,24,25,26].

Let $L^{i}=\left[c_{0}^{i},c_{2}^{i},\ldots ,c_{m_{i}-1}^{i}\right]$ be the list of chunk candidates for chunk *i*, $0<m_{i}\leq 2^{w}$. Let $c_{j_{0}}^{i_{0}},\ldots ,c_{j_{n}}^{i_{n}}$ be chunk candidates $0\leq i_{0}<\cdots <i_{n}<N,0\leq j_{i}<m_{i}$. The function $\mathrm{combine}(c_{j_{0}}^{i_{0}},\ldots ,c_{j_{n}}^{i_{n}})$ returns a new chunk candidate c such that $c=(c_{j_{0}}^{i_{0}}.score+\ldots +c_{j_{n}}^{i_{n}}.score,c_{j_{0}}^{i_{0}}.value\|\ldots \|c_{j_{n}}^{i_{n}}.value).$ Note that when $i_{0}=0,i_{1}=1,\ldots ,i_{N-1}=N-1$, c will be a full key candidate.

**Definition** **1.** *The key enumeration problem entails traversing the N lists $L^{i}$, 0≤i<N, while picking a chunk candidate $c_{j_{i}}^{i}$ from each $L^{i}$ to generate full key candidates c = combine (c${}_{j_{0}}^{i_{0}},\ldots ,$c${}_{j_{n}}^{i_{n}}$). Moreover, we call an algorithm generating full key candidates* *c* *a key enumeration algorithm (KEA).*

Note that the key enumeration problem has been stated in a general way; however, there are many other variants to this problem. These variants relate to the manner in which the key candidates are generated by a key enumeration algorithm.

A different version of the key enumeration problem is enumerating key candidates c such that their total accumulated scores follow a specific order. For example, for many side-channel scenarios, it is necessary to enumerate key candidates c starting at the one having the highest score, followed by the one having the second highest score, and so on. In these scenarios, we need a key enumeration algorithm to enumerate high-scoring key candidates in decreasing order based on their total accumulated scores. For example, such an algorithm would allow us to find the top *M* highest scoring candidates in decreasing order, where $1\leq M\ll 2^{W}$. Furthermore, such an algorithm is known as an optimal key enumeration algorithm.

Another version of the same problem is enumerating all the key candidates c such that their total accumulated scores satisfy a specified property rather than a specific order. For example, for some side-channel scenarios, it would be useful to enumerate all key candidates of which their total accumulated scores lie in an interval $\left[B_{1},B_{2}\right]$. In these scenarios, the key enumeration algorithm has to enumerate all key candidates of which their total accumulated scores lie in that interval, however such enumeration may be not performed in a specified order; still, it does need to ensure that all fitting key candidates will be generated once it has completed. This is, the algorithm will generate all the key candidates of which their total accumulated scores satisfy the condition in any order. Such an algorithm would allow us to find the top *M* highest scoring candidates in any order if the interval is well defined, for example. Moreover, such an algorithm is commonly known as a nonoptimal key enumeration algorithm.

We note that the key enumeration problem arises in other contexts. For example, in the area of statistical cryptanalysis. In particular, the problem of merging two lists of subkey candidates was encountered by Junod and Vaudenay [27]. The small cardinality of the lists ($2^{13}$) was such that the simple approach that consists of merging and sorting the lists of subkeys was tractable. Another related problem is list decoding of convolutional codes by means of the Viterbi algorithm [28]. However, such algorithms are usually designed to output a small number of most likely candidates determined a priori, whilst our aim is at algorithms able to perform long enumerations, i.e., only those key enumeration algorithms designed to be able to perform enumerations of $2^{30}$ or more key candidates.

## 4. Key Enumeration Algorithms

In this section, we review several key enumeration algorithms. Since our target is algorithms able to perform long enumerations, our review procedure consisted of examining only those research works presenting key enumeration algorithms designed to be able to perform enumerations of $2^{30}$ or more key candidates. Basically, we reviewed research proposals mainly from the side-channel literature methodically and in detail, starting from the research paper by Veyrat-Charvillon et al. [18], which was the first to look closely at the conquer part in side-channel analysis with the goal of testing several billions of key candidates. Particularly, its authors noted that none of the key enumeration algorithms proposed in the research literature until then were scalable, requiring novel algorithms to tackle the problem. Hence, they presented an optimal key enumeration algorithm that has inspired other more recent proposals.

Broadly speaking, optimal key enumeration algorithms [18,28] tend to consume more memory and to be less efficient while generating high-scoring key candidates, whereas nonoptimal key enumeration algorithms [12,13,14,15,16,17,26,29] are expected to run faster and to consume less memory. Table 1 shows a preliminary taxonomy of the key enumeration algorithms to be reviewed in this section. Each algorithm will be detailed and analyzed below according to its overall performance, scalability, and memory consumption.

### 4.1. An Optimal Key Enumeration Algorithm

We study the optimal key enumeration algorithm (OKEA) that was introduced in the research paper [18]. We will firstly give the basic idea behind the algorithm by assuming the encoding of the secret key is represented as two chunks; hence, we have access to two lists of chunk candidates.

#### 4.1.1. Setup

Let $L^{0}=\left[c_{0}^{0},c_{1}^{0},\ldots ,c_{m_{0}-1}^{0}\right]$ and $L^{1}=\left[c_{0}^{1},c_{1}^{1},\ldots ,c_{m_{1}-1}^{1}\right]$ be the two lists respectively. Each list is in decreasing order based on the score component of its chunk candidates. Let us define an extended candidate as a 4-tuple of the form $C:=(c_{j_{0}}^{0},c_{j_{1}}^{1},j_{0},j_{1})$ and its score as $c_{j_{0}}^{0}.score+c_{j_{1}}^{1}.score$. Additionally, let Q be a priority queue that will store extended candidates in decreasing order based on their score.

This data structure Q supports three methods. Firstly, the method Q.poll() retrieves and removes the head from this queue Q or returns null if this queue is empty. Secondly, the method Q.add(e) inserts the specified element *e* into the priority queue Q. Thirdly, the method Q.clear() removes all the elements from the queue Q. The queue will be empty after this method returns. By making use of a heap, we can support any priority-queue operation on a set of size n in $O(log_{2}\left(n\right))$ time.

Furthermore, let X and Y be two vectors of bits that grow as needed. These are employed to track an extended candidate C in Q. C is in Q only if both $X_{j_{0}}$ and $Y_{j_{1}}$ are set to 1. By default, all bits in a vector initially have the value 0.

#### 4.1.2. Basic Algorithm

At the initial stage, queue Q will be created. Next, the extended candidate $\left(c_{0}^{0},c_{0}^{1},0,0\right)$ will be inserted into the priority queue and both $X_{0}$ and $Y_{0}$ will be set to 1. In order to generate a new key candidate, the routine nextCandidate, defined in Algorithm 1, should be executed.

Let us assume that $m_{0},m_{1}>1$. First, the extended candidate $\left(c_{0}^{0},c_{0}^{1},0,0\right)$ will be retrieved and removed from Q, and then, $X_{0}$ and $Y_{0}$ will be set to 0. The two if blocks of instructions will then be executed, meaning that the extended candidates $\left(c_{1}^{0},c_{0}^{1},1,0\right)$ and $\left(c_{0}^{0},c_{1}^{1},0,1\right)$ will be inserted into Q. Moreover, the entries $X_{0},X_{1},Y_{0}$, and $Y_{1}$ will be set to 1, while the other entries of X and Y will remain as 0. The routine nextCandidate will then return $c_{0,0}=combine\left(c_{0}^{0},c_{0}^{1}\right)$, which is the highest score key candidate, since $L^{0}$ and $L^{1}$ are in decreasing order. At this point, the two extended candidates $\left(c_{1}^{0},c_{0}^{1},1,0\right)$ and $\left(c_{0}^{0},c_{1}^{1},0,1\right)$ (both in Q) are the only ones that can have the second highest score. Therefore, if Algorithm 2 is called again, the first instruction will retrieve and remove the extended candidate with the second highest score, say $\left(c_{0}^{0},c_{1}^{1},0,1\right)$, from Q and then the second instruction will set $X_{0}$ and $Y_{1}$ to 0. The first if condition will be attempted, but this time, it will be false since $X_{1}$ is set to 1. However, the second if condition will be satisfied, and therefore, $\left(c_{0}^{0},c_{2}^{1},0,2\right)$ will be inserted into Q and the entries $X_{0}$ and $Y_{2}$ will be set to 1. The method will then return $c_{0,1}=\mathrm{combine}\left(c_{0}^{0},c_{1}^{1}\right)$, which is the second highest score key candidate.

**Algorithm 1** outputs the next highest-scoring key candidate from $L^{0}$ and $L^{1}$.
1:**function**NextCandidate(Q)2:    $\left(c_{j_{0}}^{0},c_{j_{1}}^{1},j_{0},j_{1}\right)\leftarrow Q.\mathrm{poll}\left(\right);$3:    $X_{j_{0}}\leftarrow 0;Y_{j_{1}}\leftarrow 0;$4:    **if** $\left(j_{0}+1\right)<L^{0}.\mathrm{size}\left(\right)$ **and**  $X_{j_{0}+1}=0$ **then**5:        $c_{j_{0}+1}^{0}\leftarrow L^{0}.\mathrm{get}\left(j_{0}+1\right)$;6:        $Q.\mathrm{add}(\left(c_{j_{0}+1}^{0},c_{j_{1}}^{1},j_{0}+1,j_{1}\right))$;7:        $X_{j_{0}+1}\leftarrow 1;Y_{j_{1}}\leftarrow 1;$8:    **end if**9:    **if** $\left(j_{1}+1\right)<L^{1}.\mathrm{size}\left(\right)$ **and**  $Y_{j_{1}+1}=0$ **then**10:        $c_{j_{1}+1}^{1}\leftarrow L^{1}.\mathrm{get}\left(j_{1}+1\right)$;11:        $Q.\mathrm{add}(\left(c_{j_{0}}^{0},c_{j_{1}+1}^{1},j_{0},j_{1}+1\right))$;12:        $X_{j_{0}}\leftarrow 1;Y_{j_{1}+1}\leftarrow 1;$13:    **end if**14:    **return** $c_{j_{0},j_{1}}=\mathrm{combine}\left(c_{j_{0}}^{0},c_{j_{1}}^{1}\right)$;15:
**end function**



At this point, the two extended candidates $\left(c_{1}^{0},c_{0}^{1},1,0\right)$ and $\left(c_{0}^{0},c_{2}^{1},0,2\right)$ (both in Q) are the only ones that can have the third highest score. As for why, we know that the algorithm has generated $c_{0,0}$ and $c_{0,1}$ so far. Since $L_{0}$ and $L_{1}$ are in decreasing order, we have that either $c_{0,0}.score\geq c_{0,1}.score\geq c_{1,0}.score\geq c_{0,2}.score$ or $c_{0,0}.score\geq c_{0,1}.score\geq c_{0,2}.score\geq c_{1,0}.score$. Also, any other extended candidate yet to be inserted into Q cannot have the third highest score for the same reason. Consider, for example, $\left(c_{1}^{0},c_{1}^{1},1,1\right)$: this extended candidate will be inserted into Q only if $\left(c_{1}^{0},c_{0}^{1},1,0\right)$ has been retrieved and removed from Q. Therefore, if Algorithm 1 is executed again, it will return the third highest scoring key candidate and have the extended candidate with the fourth highest score placed at the head of Q. In general, the manner in which this algorithm travels through the $m_{0}\times m_{1}$ matrix of key candidates guarantees to output key candidates in a decreasing order based on their total accumulated score, i.e., this algorithm is an optimal key enumeration algorithm.

Regarding how fast queue Q grows, let $N_{Q}^{s}$ be the number of extended candidates in Q after the function nextCandidate has been called s≥0 times. Clearly, we have that $N_{Q}^{0}$ = 1, since Q only contains the extended candidate $\left(c_{0}^{0},c_{0}^{1},0,0\right)$ after initialisation. Also, $N_{Q}^{m_{1}\cdot m_{2}}=0$ because, after $m_{1}\cdot m_{2}$ calls to the function, there will be no more key candidates to be enumerated. Note that, during the execution of the function nextCandidate, an extended candidate will be removed from Q and two new extended candidates might be inserted into Q. Considering the way in which an extended candidate is inserted into the queue, Q may contain at most one element in each row and column at any stage; hence, $N_{Q}^{s}\leq \min\left(m_{0},m_{1}\right)$ for 0≤s≤m1·m2.

#### 4.1.3. Complete Algorithm

**Figure 1:**
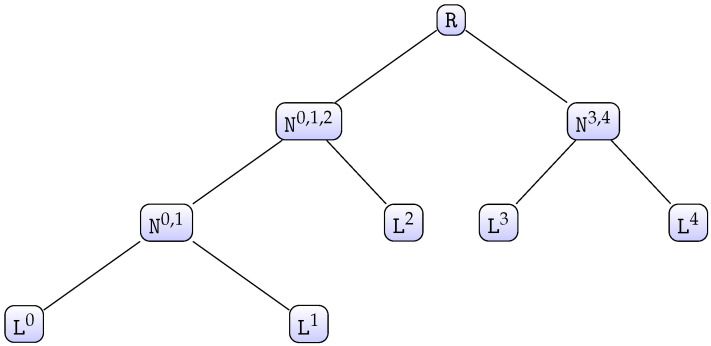
Binary tree built from $L^{i},0\leq i<5$.

Note that Algorithm 1 works properly if both input lists are in decreasing order. Hence, it may be generalized to a number of lists greater than 2 by employing a divide-and-conquer approach, which works by recursively breaking down the problem into two or more subproblems of the same or related type until these become simple enough to be solved directly. The solutions to the subproblems are then combined to give a solution to the original problem [30]. To explain the complete algorithm, let us consider the case when there are five chunks as an example. We have access to five lists of chunk candidates $L^{i},0\leq i<5$, each of which has a size of $m_{i}$. We first call initialise(0,4), as defined in Algorithm 2. This function will build a tree-like structure from the five given lists (see Figure 1).

Each node $N^{i,\ldots ,f}$ is a 6-tuple of the form $\left(N^{i,\ldots ,q},N^{q+1,\ldots ,f},Q^{i,\ldots ,f},X^{i,\ldots ,f},Y^{i,\ldots ,f},L^{i,\ldots ,f}\right)$, where $N^{i,\ldots ,q}$ and $N^{q+1,\ldots ,f}$ are the children nodes, $Q^{i,\ldots ,f}$ is a priority queue, $X^{i,\ldots ,f}$ and $Y^{i,\ldots ,f}$ are bit vectors, and $L^{i,\ldots ,f}$ a list of chunk candidates. Additionally, this data structure supports the method size(), which returns the maximum number of chunk candidates that this node can generate. This method is easily defined in a recursive way: if $N^{i,\ldots ,f}$ is a leaf node, then the method will return $L^{i,\ldots ,f}.\mathrm{size}\left(\right)$ or else, the method will return $N^{i,\ldots ,q}.\mathrm{size}\left(\right)\times N^{q+1,\ldots ,f}.\mathrm{size}\left(\right)$. To avoid computing this value each time this method is called, a node will internally store the value once it has been computed for the first time. Hence, the method will only return the stored value from the second call onwards. Furthermore, the function $\mathrm{getCandidate}(N^{i,\ldots ,f},j)$, as defined in Algorithm 3, returns the $j_{th}$ best chunk candidate (chunk candidate of which its score rank is *j*) from the node $N^{i,\ldots ,f}.$

In order to generate the first *N* best key candidates from the root node R, with $R:=N^{0,\ldots ,4}$, we simply run nextCandidate(R), as defined in Algorithm 4, *N* times. This function internally calls the function getCandidate with suitable parameters each time it is required. Calling $\mathrm{getCandidate}(N^{i,\ldots ,f},j)$ may cause this function to internally invoke $\mathrm{nextCandidate}(N^{i,\ldots ,f})$ to generate ordered key candidates from the inner node $N^{i,\ldots ,f}$ on the fly. Therefore, any inner node $N^{i,\ldots ,f}$ should keep track of the chunk candidates returned by $\mathrm{getCandidate}(N^{i,\ldots ,f},j)$ when called by its parent; otherwise, the *j* best chunk candidates from $N^{i,\ldots ,f}$ would have to be generated each time such a call is done, which is inefficient. To keep track of the returned chunk candidates, each node $N^{i,\ldots ,f}$ updates its internal list $L^{i,\ldots ,f}$ (see lines 5 to 7 in Algorithm 3).

**Algorithm 2** creates and initialises each node of the tree-like structure.
1:**function**initialise(i,f)2:    **if** f=i **then**3:        $L^{i}\leftarrow \left(\mathrm{null},\mathrm{null},\mathrm{null},\mathrm{null},\mathrm{null},L^{i}\right);$4:        **return** $L^{i}$;5:    **else**6:        $q\leftarrow \lfloor \frac{i+f}{2}\rfloor$;7:        $N^{i,\ldots ,q}\leftarrow \mathrm{initialise}\left(i,q\right)$;8:        $N^{q+1,\ldots ,f}\leftarrow \mathrm{initialise}\left(q+1,f\right)$;9:        $c_{0}^{i,\ldots ,q}\leftarrow \mathrm{getCandidate}\left(N^{i,\ldots ,q},0\right)$;10:        $c_{0}^{q+1,\ldots ,f}\leftarrow \mathrm{getCandidate}\left(N^{q+1,\ldots ,f},0\right)$;11:        $Q^{i,\ldots ,f}.\mathrm{add}\left(\left(c_{0}^{i,\ldots ,q},c_{0}^{q+1,\ldots ,f},0,0\right)\right)$;12:        $X_{0}^{i,\ldots ,f}\leftarrow 1$; $Y_{0}^{i,\ldots ,f}\leftarrow 1;$13:        $N^{i,\ldots ,f}\leftarrow \left(N^{i,\ldots ,q},N^{q+1,\ldots ,f},Q^{i,\ldots ,f},X^{i,\ldots ,f},Y^{i,\ldots ,f},L^{i,\ldots ,f}\right);$14:        **return** $N^{i,\ldots ,f}$;15:    **end if**16:
**end function**



**Algorithm 3** outputs the $j_{th}$ best chunk candidate from the node $N^{i,\ldots ,f}$.
1:**function**getCandidate($N^{i,\ldots ,f},j$)2:    **if** $N^{i,\ldots ,f}$ is a leaf **then**3:        **return** $L^{i,\ldots ,f}.\mathrm{get}\left(j\right)$;4:    **end if**5:    **if** $j\geq L^{i,\ldots ,f}.\mathrm{size}\left(\right)$ **then**6:        $L^{i,\ldots ,f}.\mathrm{add}\left(\mathrm{nextCandidate}\left(N^{i,\ldots ,f}\right)\right)$;7:    **end if**8:    **return** $L^{i,\ldots ,f}.\mathrm{get}\left(j\right)$;9:
**end function**



**Algorithm 4** outputs the next highest-scoring chunk candidate from the node $N^{i,\ldots ,f}$.
1:**function**nextCandidate($N^{i,\ldots ,f}$)2:    $\left(c_{j_{x}}^{x},c_{j_{y}}^{y},j_{x},j_{y}\right)\leftarrow Q^{i,\ldots ,f}.\mathrm{poll}\left(\right);$(x={i,…,q},y={q+1,…,f}).3:    $X_{j_{x}}^{i,\ldots ,f}\leftarrow 0;Y_{j_{y}}^{i,\ldots ,f}\leftarrow 0;$4:    **if** $\left(j_{x}+1\right)<N^{i,\ldots ,q}.\mathrm{size}\left(\right)$ **and**  $X_{j_{x}+1}^{i,\ldots ,f}=0$ **then**5:        $c_{j_{x}+1}^{x}\leftarrow \mathrm{getCandidate}\left(N^{i,\ldots ,q},j_{x}+1\right);$6:        $Q^{i,\ldots ,f}.\mathrm{add}\left(\left(c_{j_{x}+1}^{x},c_{j_{y}}^{y},j_{x}+1,j_{y}\right)\right);$7:        $X_{j_{x}+1}^{i,\ldots ,f}\leftarrow 1;Y_{j_{y}}^{i,\ldots ,f}\leftarrow 1;$8:    **end if**9:    **if** $\left(j_{y}+1\right)<N^{q+1,\ldots ,f}.\mathrm{size}\left(\right)$ **and**  $Y_{j_{y}+1}^{i,\ldots ,f}=0$ **then**10:        $c_{j_{y}+1}^{y}\leftarrow \mathrm{getCandidate}\left(N^{q+1,\ldots ,f},j_{y}+1\right);$11:        $Q^{i,\ldots ,f}.\mathrm{add}\left(\left(c_{j_{x}}^{x},c_{j_{y}+1}^{y},j_{x},j_{y}+1\right)\right)$;12:        $X_{j_{x}}^{i,\ldots ,f}\leftarrow 1;Y_{j_{y}+1}^{i,\ldots ,f}\leftarrow 1;$13:    **end if**14:    **return** $\mathrm{combine}(c_{j_{x}}^{x},c_{j_{y}}^{y});$15:
**end function**



#### 4.1.4. Memory Consumption

Let us suppose that the encoding of a secret key is $W=2^{a+b}$ bits in size and that we set $w=2^{a}$; therefore, $N=2^{b}$. Hence, we have access to N lists $L^{i}$, $0\leq i<2^{b}$, each of which has $m_{i}$ chunk candidates. Suppose we would like to generate the first *N* best key candidates. We first invoke initialise(0,N−1) (Algorithm 2). This call will create a tree-like structure with b+1 levels starting at 0.

- The root node $R:=N^{0,\ldots ,2^{b}-1}$ at level 0.
- The inner nodes $N^{I_{d}}:=N_{\lambda }^{i_{d}}$ with $I_{d}=\left\{i_{d}\cdot 2^{b-\lambda },\ldots ,\left(i_{d}+1\right)\cdot 2^{b-\lambda }-1\right\}$, where λ,0<λ<b, is the level and $i_{d},0\leq i_{d}<2^{\lambda },$ is the node identification at level λ.
- The leaf nodes $L^{i}$ at level *b* for $0\leq i<2^{b}$.

This tree will have $2^{0}+2^{1}+\cdots +2^{b}=2^{b+1}-1$ nodes.

Let $M_{k}$ be the number of bits consumed by chunk candidates stored in memory after calling the function nextCandidate with R as a parameter *k* times. A chunk candidate at level 0≤λ≤b is of the form $\left(score,\left[e_{0},\ldots ,e_{2^{b-\lambda }-1}\right]\right)$ with score being a real number and $e_{l}$ being bit strings. Let $B_{\lambda }$ be the number of bits a chunk candidate at level λ occupies in memory.

First note that invoking initialise(0,N−1) causes each internal node’s list to grow, since

1. At creation of nodes $L^{i}$ (lines 2 to 4), $L^{i}$ is created by setting $L^{i}$’s internal list to $L^{i}$ and by setting $L^{i}$’s other components to null.
2. At creation of both R and nodes $N_{\lambda }^{i_{d}}$, for 0<λ<b−1 and $0\leq i_{d}<2^{\lambda }$, the execution of the function getCandidate (lines 9 to 10) makes their corresponding left child (right child) store a new chunk candidate in their corresponding internal list. That is, for $0<\lambda \leq b-1,0\leq id<2^{\lambda }$, the $N_{\lambda }^{id}$’s internal list has a new element.

Therefore, $M_{0}=\sum_{\lambda =1}^{b-1}2^{\lambda }B_{\lambda }+B_{b}\left(\sum_{i=0}^{2^{b}-1}m_{i}\right).$

Suppose the best key candidate is about to be generated, then nextCandidate(R) will be executed for the first time. This routine will remove the extended candidate $\left(c_{0}^{x},c_{0}^{y},0,0\right)$ out of R’s priority queue. If it enters the first **if** (lines 4 to 8), it will make the call $\mathrm{getCandidate}(N_{1}^{0},1)$ (line 5), which may cause each node, except for the leaf nodes, of the left sub-tree to store at most a new chunk candidate in its corresponding internal list. Hence, retrieving the chunk candidate $c_{1}^{x}$ may cause at most $2^{\lambda -1}$ chunk candidates per level λ,1≤λ<b, to be stored. Likewise, if it enters the second **if** (lines 9 to 13), it will call the function $\mathrm{getCandidate}(N_{1}^{1},1)$ (line 10), which may cause each node, except for the leaf nodes, of the right sub-tree to store at most a new chunk candidate in its corresponding internal list. Therefore, retrieving the chunk candidate $c_{1}^{y}$ (line 10) may cause at most $2^{\lambda -1}$ chunk candidates per level λ,1≤λ<b, to be stored. Therefore, after generating the best key candidate, $p_{\lambda }^{\left(1\right)}\leq 2^{\lambda }$ chunk candidates per level λ,1≤λ<b, will be stored in memory; hence, $M_{0}\leq M_{1}=M_{0}+\sum_{\lambda =1}^{b-1}p_{\lambda }^{\left(1\right)}B_{\lambda }\leq 2\sum_{\lambda =1}^{b-1}2^{\lambda }B_{\lambda }+B_{b}\left(\sum_{i=0}^{2^{b}-1}m_{i}\right)$ bits are consumed by chunk candidates stored in memory.

Let us assume that k−1 key candidates have already been generated; therefore, $M_{k-1}$ bits are consumed by chunk candidates in memory, with $M_{k-1}=M_{0}+\sum_{d=1}^{k-1}\sum_{\lambda =1}^{b-1}p_{\lambda }^{\left(d\right)}B_{\lambda }$. Let us now suppose the $k_{th}$ best key candidate is about to be generated; then, the method nextCandidate(R) will be executed for the $k_{th}$ time. This routine will remove the best extended candidate $\left(c_{j_{x}}^{x},c_{j_{y}}^{y},j_{x},j_{y}\right)$ out of the R’s priority queue. It will then attempt to insert two new extended candidates into R’s priority queue. As seen previously, retrieving the chunk candidate $c_{j_{x}+1}^{x}$ may cause at most $2^{\lambda }-1$ chunk candidates per level λ,1≤λ<b, to be stored. Likewise, retrieving the chunk candidate $c_{j_{y}+1}^{y}$ may also cause at most $2^{\lambda -1}$ chunk candidates per level λ,1≤λ<b, to be stored. Therefore, after generating the $k_{th}$ best key candidate, $p_{\lambda }^{\left(k\right)}\leq 2^{\lambda }$ chunk candidates per level λ,1≤λ<b, will be stored in memory; hence,

$$M_{k}=M_{k-1}+\sum_{\lambda =1}^{b-1}p_{\lambda }^{\left(k\right)}B_{\lambda }=M_{0}+\sum_{d=1}^{k}\sum_{\lambda =1}^{b-1}p_{\lambda }^{\left(d\right)}B_{\lambda }$$

bits are consumed by chunk candidates stored in memory.

It follows that, if *N* key candidates are generated, then

$$M_{N}=M_{0}+\sum_{d=1}^{N}\sum_{\lambda =1}^{b-1}p_{\lambda }^{\left(d\right)}B_{\lambda }=\sum_{\lambda =1}^{b-1}2^{\lambda }B_{\lambda }+B_{b}\left(\sum_{i=0}^{2^{b}-1}m_{i}\right)+\sum_{d=1}^{N}\sum_{\lambda =1}^{b-1}p_{\lambda }^{\left(d\right)}B_{\lambda },$$

bits are consumed by chunk candidates stored in memory in addition to the extended candidates stored internally in the priority queue of the nodes R and $N_{\lambda }^{i_{d}}.$ Therefore, this algorithm may consume a large amount of memory if it is used to generate a large number of key candidates, which may be problematic.

### 4.2. A Bounded-Space Near-Optimal Key Enumeration Algorithm

We next will describe a key enumeration algorithm introduced in the research paper [13]. This algorithm builds upon OKEA and can enumerate a large number of key candidates without exceeding the available space. The trade-off is that the enumeration order is only near-optimal rather than optimal as it is in OKEA. We firstly will give the basic idea behind the algorithm by assuming the encoding of the secret key is represented as two chunks; hence, we have access to two lists of chunk candidates.

### 4.3. Basic Algorithm

**Figure 2:**
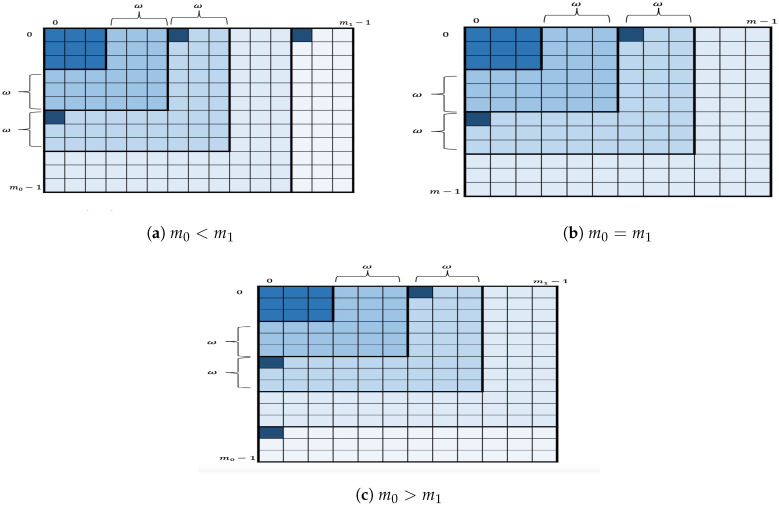
Geometric representation of the key space divided into layers of width ω=3.

Let $L^{0}=\left[c_{0}^{0},c_{1}^{0},\ldots ,c_{m_{0}-1}^{0}\right]$ and $L^{1}=\left[c_{0}^{1},c_{1}^{1},\ldots ,c_{m_{1}-1}^{1}\right]$ be the two lists, and let ω>0 be an integer such that $\omega |m_{0}$ and $\omega |m_{1}$. Each list is in decreasing order based on the score component of its chunk candidates. Let us set $m_{min}=\min\left(m_{0},m_{1}\right)$ and define $R_{k_{0},k_{1}}$ as

$$R_{k_{0},k_{1}}:=\left\{0,\ldots ,k_{0}\cdot \omega -1\right\}\times \left\{0,\ldots ,k_{1}\cdot \omega -1\right\},$$

where $k_{0},k_{1}$ are positive integers. The key space is divided into layers $layer_{k}^{\omega }$ of width ω. Figure 2 depicts each layer as a different shade of blue. Formally,

$$layer_{k}^{\omega }:=\left\{\left(c_{j_{0}}^{0},c_{j_{1}}^{1}\right)|\left(j_{0},j_{1}\right)\in R_{k,k}\setminus R_{k-1,k-1}\right\},$$

for $1\leq k\leq \frac{mmin}{w}.$ The remaining layers are defined as follows.

If $m_{0}\geq m_{1}$,

$$layer_{k}^{\omega }:=\left\{\left(c_{j_{0}}^{0},c_{j_{1}}^{1}\right)|\left(j_{0},j_{1}\right)\in R_{k,\frac{m_{min}}{\omega }}\setminus R_{k-1,\frac{m_{min}}{\omega }}\right\},$$

for $\frac{m_{min}}{\omega }<k\leq \frac{m_{0}}{\omega }$ or else

$$layer_{k}^{\omega }:=\left\{\left(c_{j_{0}}^{0},c_{j_{1}}^{1}\right)|\left(j_{0},j_{1}\right)\in R_{\frac{m_{min}}{\omega },k}\setminus R_{\frac{m_{min}}{\omega },k-1}\right\},$$

for $\frac{m_{min}}{\omega }<k\leq \frac{m_{1}}{\omega }$.

The ω-layer key enumeration algorithm: Divide the key space into layers of width ω. Then, go over $layer_{k}^{\omega }$, one by one, in increasing order. For each $layer_{k}^{\omega }$, enumerate its key candidates by running OKEA within the layer $layer_{k}^{\omega }$. More specifically, for each $layer_{k}^{\omega },1\leq k\leq \frac{m_{min}}{\omega },$ the algorithm inserts the two corners, i.e., the extended candidates $\left(c_{(k-1)\cdot \omega }^{0},c_{0}^{1},\left(k-1\right)\cdot \omega ,0\right),\left(c_{0}^{0},c_{(k-1)\cdot \omega }^{1},0,\left(k-1\right)\cdot \;\omega \right)$, into the data structure Q. The algorithm then proceeds to extract extended candidates and to insert their successors as usual but limits the algorithm to not exceed the boundaries of the layer $layer_{k}^{\omega }$ when selecting components of candidates. For the remaining layers, if any, the algorithm inserts only one corner, either the extended candidate $\left(c_{(k-1)\cdot \omega }^{0},c_{0}^{1},\left(k-1\right)\cdot \omega ,0\right)$ or the extended candidate $\left(c_{0}^{0},c_{(k-1)\cdot \omega }^{1},0,\left(k-1\right)\cdot \omega \right)$, into the data structure Q and then proceeds as usual while not exceeding the boundaries of the layer. Figure 2 also shows the extended candidates (represented as the smallest squares in a strong shade of blue within a layer) to be inserted into Q when a certain layer will be enumerated.

### 4.4. Complete Algorithm

When the number of chunks is greater than 2, the algorithm applies a recursive decomposition of the problem (similar to OKEA). Whenever a new chunk candidate is inserted into the candidate set, its value is obtained by applying the enumeration algorithm to the lower level. We explain an example to give an idea of the general algorithm. Let us suppose the encoding of the secret key is divided into 4 chunks; then, we have access to 4 lists of chunk candidates, each of which is of size $m_{i}$ with $\omega |m_{i}.$

To generate key candidates, we need to generate the two lists of chunk candidates for the lower level $L^{0,1}$ and $L^{2,3}$ on the fly as far as required. For this, we maintain a set of next potential candidates, for each dimension, $Q^{0,1}$ and $Q^{2,3}$, so that each next chunk candidate obtained from $Q^{0,1}$ (or $Q^{2,3}$) is stored in the list $L^{0,1}$(or $L^{2,3}$). Because the enumeration is performed by layers, the sizes of the data structures $Q^{1,2}$ and $Q^{3,4}$ are bounded by 2·ω. However, this is not the case for the lists $L^{0,1}$ and $L^{2,3}$, which grow as the number of candidates enumerated grows, hence becoming problematic as seen in Section 4.1.4.

**Figure 3:**
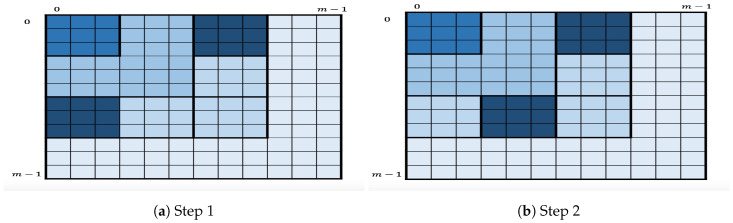
Geometric representation of the key enumeration within $layer_{3}^{3}.$

To handle this, each $layer_{k}^{\omega }$ is partitioned into squares of size ω×ω. The algorithm still enumerates the key candidates in $layer_{1}^{\omega }$ first, then in $layer_{2}^{\omega }$, and so on, but in each $layer_{k}^{\omega }$, the enumeration will be square-by-square. Figure 3 depicts the geometric representation of the key enumeration within $layer_{3}^{3},$ where a square (strong shade of blue) within a layer represents the square being processed by the enumeration algorithm. More specifically, for given nonnegative integers *I* and J, let us define $S_{I,J}^{w}$ as

$$S_{I,J}^{\omega }:=\left\{\left(c_{j_{x}},c_{j_{y}}\right)|I\cdot \omega \leq j_{x}<\left(I+1\right)\cdot \omega ,J\cdot \omega \leq j_{y}<\left(J+1\right)\cdot \omega \right\}.$$

Let us set $m_{min}=\min\left(m_{0}\cdot m_{1},m_{2}\cdot m_{3}\right)$; hence,

$$layer_{k}^{\omega }=S_{k-1,0}^{\omega }\cup S_{k-1,1}^{\omega }\cup \cdots \cup S_{k-1,k-1}^{\omega }\cup S_{k-2,k-1}^{\omega }\cup \cdots \cup S_{0,k-1}^{\omega },$$

for $1\leq k\leq \frac{m_{min}}{\omega }$. The remaining layers, if any, are also partitioned in a similar way.

The in-layer algorithm then proceeds as follows. For each $layer_{k}^{\omega },1\leq k\leq \frac{m_{min}}{\omega },$ the in-layer algorithm first enumerates the candidates in the two corner squares $S=S_{k-1,0}^{\omega }\cup S_{0,k-1}^{\omega }$ by applying OKEA on *S*. At some point, one of the two squares is completely enumerated. Assume this is $S_{k-1,0}^{\omega }$. At this point, the only square that contains the next key candidates after $S_{k-1,0}^{\omega }$ is the successor $S_{k-1,1}^{\omega }$. Therefore, when one of the squares is completely enumerated, its successor is inserted in *S*, as long as *S* does not contain a square in the same row or column. For the remaining layers, if any, the in-layer algorithm first enumerates the candidates in the square $S=S_{k-1,0}^{\omega }$ (or $S_{0,k-1}^{\omega }$) by applying OKEA on it. Once the square is completely enumerated, its successor is inserted in *S*, and so on. This in-layer partition into squares reduces the space complexity, since instead of storing the full list of chunk candidates of the lower levels, only the relevant chunk candidates are stored for enumerating the two current squares.

Because this in-layer algorithm enumerates at most two squares at any time in a layer, the tree-like structure is no longer a binary tree. A node $N^{i,\ldots ,f}$ is now extended to an 8-tuple of the form $\left(N_{0}^{i,\ldots ,q},N_{0}^{q+1,\ldots ,f},N_{1}^{i,\ldots ,q},N_{1}^{q+1,\ldots ,f},Q^{i,\ldots ,f},X^{i,\ldots ,f},Y^{i,\ldots ,f},L^{i,\ldots ,f}\right)$, where $N_{b}^{i,\ldots ,q}$ and $N_{b}^{q+1,\ldots ,f}$ for b=0,1 are the children nodes used to enumerate at most two squares in a particular layer, $Q^{i,\ldots ,f}$ is a priority queue, $X^{i,\ldots ,f}$ and $Y^{i,\ldots ,f}$ are bit vectors, and $L^{i,\ldots ,f}$ is a list of chunk candidates. Hence, the function that initialises the tree-like structure is adjusted to create the two additional children for a given node (see Algorithm 5).

**Algorithm 5** creates and initialises each node of the tree-like structure.
1:**function**initialise(i,f)2:    **if** f=i **then**3:        $L^{i}\leftarrow \left(\mathrm{null},\mathrm{null},\mathrm{null},\mathrm{null},\mathrm{null},\mathrm{null},\mathrm{null},L^{i}\right);$4:        **return** $L^{i}$;5:    **else**6:        $q\leftarrow \lfloor \frac{i+f}{2}\rfloor$;7:        $N_{0}^{i,\ldots ,q}\leftarrow \mathrm{initialise}\left(i,q\right)$;8:        $N_{0}^{q+1,\ldots ,f}\leftarrow \mathrm{initialise}\left(q+1,f\right)$;9:        $N_{1}^{i,\ldots ,q}\leftarrow \mathrm{initialise}\left(i,q\right)$;10:        $N_{1}^{q+1,\ldots ,f}\leftarrow \mathrm{initialise}\left(q+1,f\right)$;11:        $c_{0}^{i,\ldots ,q}\leftarrow \mathrm{getCandidate}\left(N_{0}^{i,\ldots ,q},0,2\right)$;12:        $c_{0}^{q+1,\ldots ,f}\leftarrow \mathrm{getCandidate}\left(N_{1}^{q+1,\ldots ,f},0,2\right)$;13:        $Q^{i,\ldots ,f}.\mathrm{add}\left(\left(c_{0}^{i,\ldots ,q},c_{0}^{q+1,\ldots ,f},0,0\right)\right)$;14:        $X_{0}^{i,\ldots ,f}\leftarrow 1$; $Y_{0}^{i,\ldots ,f}\leftarrow 1$;15:        $N^{i,\ldots ,f}\leftarrow \left(N_{0}^{i,\ldots ,q},N_{0}^{q+1,\ldots ,f},N_{1}^{i,\ldots ,q},N_{1}^{q+1,\ldots ,f},Q^{i,\ldots ,f},X^{i,\ldots ,f},Y^{i,\ldots ,f},L^{i,\ldots ,f}\right)$;16:        **return** $N^{i,\ldots ,f}$;17:    **end if**18:
**end function**



**Algorithm 6** outputs the $j_{th}$ chunk candidate from the node $N^{i,\ldots ,f}$.
1:**function**getCandidate($N^{i,\ldots ,f},j,sw$)2:    **if** $N^{i,\ldots ,f}$ is a leaf **then**3:        **return** $L^{i,\ldots ,f}.\mathrm{get}\left(j\right)$;4:    **end if**5:    **if** sw=0 **then**6:        $\mathrm{restart}(N^{i,\ldots ,f})$;7:    **else**8:        **if** sw=1 **then**9:           $L^{i,\ldots ,f}.\mathrm{clear}\left(\right)$;10:        **end if**11:    **end if**12:    j←jmodω;13:    **if** $j\geq L^{i,\ldots ,f}.\mathrm{size}\left(\right)$ **then**14:        $L^{i,\ldots ,f}.\mathrm{add}\left(\mathrm{nextCandidate}\left(N^{i,\ldots ,f}\right)\right)$;15:    **end if**16:    **return** $L^{i,\ldots ,f}.\mathrm{get}\left(j\right)$;17:
**end function**



**Algorithm 7** outputs the next chunk candidate from the node $N^{i,\ldots ,f}$.
1:**function**nextCandidate($N^{i,\ldots ,f}$)2:    $\left(c_{j_{x}}^{x},c_{j_{y}}^{y},j_{x},j_{y}\right)\leftarrow Q^{i,\ldots ,f}.\mathrm{poll}\left(\right);$(x={i,…,q},y={q+1,…,f})3:    $X_{j_{x}}^{i,\ldots ,f}\leftarrow 0;Y_{j_{y}}^{i,\ldots ,f}\leftarrow 0;$4:    $I\leftarrow \left\lfloor \frac{j_{x}}{\omega }\right\rfloor ;J\leftarrow \left\lfloor \frac{j_{y}}{\omega }\right\rfloor ;b=\left(I\geq J\right)?0:1;$5:    **if** $S_{I,J}$ is completely enumerated **then**6:        $last_{I}\leftarrow N_{0}^{i,\ldots ,q}.\mathrm{size}\left(\right)/\omega -1$;7:        $last_{J}\leftarrow N_{1}^{q+1,\ldots ,f}.\mathrm{size}\left(\right)/\omega -1$;8:        **if** I=J **or** ($I>last_{J}$ **and** $J=last_{J}$) **or** ($J>last_{I}$ **and**$I=last_{I}$) **then**9:           **if** $\left(j_{x}+1\right)<\left(last_{I}+1\right)\cdot \omega$ **then**10:               $c_{j_{x}+1}^{x}\leftarrow \mathrm{getCandidate}\left(N_{0}^{i,\ldots ,q},j_{x}+1,1\right);$11:               $c_{0}^{y}\leftarrow \mathrm{getCandidate}\left(N_{0}^{q+1,\ldots ,f},0,0\right);$12:               $Q^{i,\ldots ,f}.\mathrm{add}\left(\left(c_{j_{x}+1}^{x},c_{0}^{y},j_{x}+1,0\right)\right);$13:               $X_{j_{x}+1}^{i,\ldots ,f}\leftarrow 1;Y_{0}^{i,\ldots ,f}\leftarrow 1;$14:           **end if**15:           **if** $\left(j_{y}+1\right)<\left(last_{J}+1\right)\cdot \omega$ **then**16:               $c_{0}^{x}\leftarrow \mathrm{getCandidate}\left(N_{1}^{i,\ldots ,q},0,0\right);$17:               $c_{j_{y}+1}^{y}\leftarrow \mathrm{getCandidate}\left(N_{1}^{q+1,\ldots ,f},j_{y}+1,1\right);$18:               $Q^{i,\ldots ,f}.\mathrm{add}\left(\left(c_{0}^{x},c_{j_{y}+1}^{y},0,j_{y}+1\right)\right);$19:               $X_{0}^{i,\ldots ,f}\leftarrow 1;Y_{j_{y}+1}^{i,\ldots ,f}\leftarrow 1;$20:           **end if**21:        **else**22:           **if** no candidates in same row/column as $\mathrm{Successor}(S_{I,J})$ **then**23:               $\left(c_{k}^{x},c_{l}^{y},k,l\right)\leftarrow \mathrm{getHighestScoreCandidate}\left(\mathrm{Successor}\left(S_{I,J}\right)\right)$;24:               $Q^{i,\ldots ,f}.\mathrm{add}\left(\left(c_{k}^{x},c_{l}^{y},k,l\right)\right);$25:               $X_{k}^{i,\ldots ,f}\leftarrow 1;Y_{l}^{i,\ldots ,f}\leftarrow 1;$26:           **end if**27:        **end if**28:    **else**29:        **if** $\left(j_{x}+1,j_{y}\right)\in S_{I,J}$ **and** $X_{j_{x}+1}^{i,\ldots ,f}$ is set to 0 **then**30:           $c_{j_{x}+1}^{x}\leftarrow \mathrm{getCandidate}\left(N_{b}^{i,\ldots ,q},j_{x}+1,2\right);$31:           $Q^{i,\ldots ,f}.\mathrm{add}\left(\left(c_{j_{x}+1}^{x},c_{j_{y}}^{y},j_{x}+1,j_{y}\right)\right);$32:           $X_{j_{x}+1}^{i,\ldots ,f}\leftarrow 1;Y_{j_{y}}^{i,\ldots ,f}\leftarrow 1;$33:        **end if**34:        **if** $\left(j_{x},j_{y}+1\right)\in S_{I,J}$ **and** $X_{j_{y}+1}^{i,\ldots ,f}$ is set to 0 **then**35:           **if** I=J **then**36:               $c_{j_{y}+1}^{y}\leftarrow \mathrm{getCandidate}\left(N_{1}^{q+1,\ldots ,f},j_{y}+1,2\right);$37:           **else**38:               $c_{j_{y}+1}^{y}\leftarrow \mathrm{getCandidate}\left(N_{b}^{q+1,\ldots ,f},j_{y}+1,2\right);$39:           **end if**40:           $Q^{i,\ldots ,f}.\mathrm{add}\left(\left(c_{j_{x}}^{x},c_{j_{y}+1}^{y},j_{x},j_{y}+1\right)\right);$41:           $X_{j_{x}}^{i,\ldots ,f}\leftarrow 1;Y_{j_{y}+1}^{i,\ldots ,f}\leftarrow 1;$42:        **end if**43:    **end if**44:    **return** $\mathrm{combine}(c_{j_{x}}^{x},c_{j_{y}}^{y})$;45:
**end function**



Moreover, the function $\mathrm{getCandidate}(N^{i,\ldots ,f},j,sw)$ is also adjusted so that each node’s internal list $L^{i,\ldots ,f}$ has at most ω chunk candidates at any stage of the algorithm (see Algorithm 6). This function internally makes the call to $\mathrm{restart}(N^{i,\ldots ,f})$ if sw=0. The call to $\mathrm{restart}(N^{i,\ldots ,f})$ causes $N^{i,\ldots ,f}$ to restart its enumeration, i.e., after $\mathrm{restart}(N^{i,\ldots ,f})$ has been invoked, calling $\mathrm{nextCandidate}(N^{i,\ldots ,f})$ will return the first chunk candidate from $N^{i,\ldots ,f}$. Also, the function $\mathrm{getHighestScoreCandidate}(S_{I,J}^{\omega })$ returns the highest-scoring extended candidate from the square $S_{I,J}^{\omega }$. Note this function is called to get the highest-scoring extended candidate from the successor of $S_{I,J}^{\omega }$. At this point, the content of the internal list of $N_{0}^{q+1,\ldots ,f}$ is cleared if b=0. Otherwise, the content of the internal list of $N_{1}^{i,\ldots ,q}$ is cleared, if b=1. Finally, Algorithm 7 precisely describes the manner in which this enumeration works.

#### 4.4.1. Parallelization

The original authors of the research paper [13] suggest having OKEA run in parallel per square within a layer, but this has a negative effect on the algorithm’s near-optimality property and even on its overall performance since there are squares within a layer that are strongly dependent on others, i.e., for the algorithm to enumerate the successor square, say, $S_{I,J+1}$ within a layer, it requires having information that is obtained during the enumeration of $S_{I,J}$. Hence, this strategy may incur extra computation and is also difficult to implement.

#### 4.4.2. Variant

**Figure 4:**
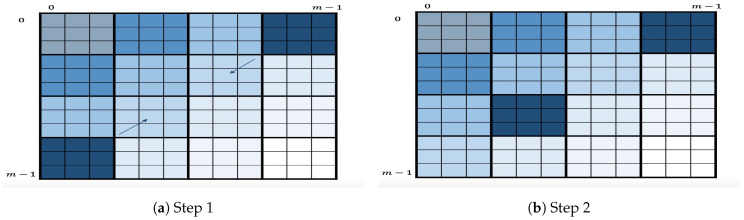
Geometric representation of the key enumeration for variant.

As a variant of this algorithm, we propose to slightly change the definition of layer. Here, a layer consists of all the squares within a secondary diagonal, as shown in Figure 4. The variant will follow the same process as the original algorithm, i.e., enumeration layer by layer starting at the first secondary diagonal. Within each layer, it will first enumerate the two square corners $S=S_{k-1,0}\cup S_{0,k-1}$ by applying OKEA on it. Once one of two squares is enumerated, let us say $S_{k-1,0}$, its successor $S_{k-2,1}$ will be inserted in *S* as long as such insertion is possible. The algorithm will continue the enumeration by applying OKEA on the updated *S* and so on. This algorithm is motivated by the intuition that enumerating secondary diagonals may improve the quality of order of output key candidates, i.e., it may be closer to optimal. This variant, however, may have a potential disadvantage in the multidimensional case because it strongly depends on having all the previously enumerated chunk candidates of both dimension *x* and *y* stored. To illustrate this, let us suppose that this square $S_{k-2,1}$ is to be inserted. Then, the algorithm needs to insert its highest-scoring extended candidate, $\left(c_{(k-2)\cdot \omega }^{x},c_{\omega }^{y},\left(k-2\right)\cdot \omega ,\omega \right)$, into the queue. Hence, the algorithm needs to somehow have both $c_{(k-2)\cdot \omega }^{x}$ and $c_{\omega }^{y}$ readily accessible when needed. This implies the need to store them when they are being enumerated (in previous layers). Comparatively, the original algorithm only requires having the ω previously generated chunk candidates of both dimension *x* and *y* stored, which is advantageous in terms of memory consumption.

### 4.5. A Simple Stack-Based, Depth-First Key Enumeration Algorithm

We next present a memory-efficient, nonoptimal key enumeration algorithm that generates key candidates of which their total scores are within a given interval $\left[B_{1},B_{2}\right]$ that is based on the algorithm introduced by Martin et al. in the research paper [16]. We note that the original algorithm is fairly efficient while generating a new key candidate; however, its overall performance may be negatively affected by its use of memory, since it was originally designed to store each new generated key candidate, each of which is tested only once the algorithm has completed the enumeration. Our variant, however, makes use of a stack (last-in-first-out queue) during the enumeration process. This helps in maintaining the state of the algorithm. Each newly generated key candidate may be tested immediately, and there is no need for candidates to be stored for future processing.

Our variant basically performs a depth-first search in an undirected graph *G* originated from the N lists of chunk candidates $L^{i}=\left[c_{0}^{i},c_{n}^{i},\ldots ,c_{m_{i}-1}^{i}\right]$. This graph *G* has $\sum_{i=0}^{N-1}m_{i}$ vertices, each of which represents a chunk candidate. Each vertex $v_{j}^{i}$ is connected to the vertices $v_{k}^{i+1},0\leq i<N-1,0\leq j<m_{i},0\leq k<m_{i+1}$. At any vertex $v_{j}^{i}$, the algorithm will check if $c_{j}^{i}.score$ plus an accumulated score is within the given interval $\left[B_{1},B_{2}\right]$. If so, it will select the chunk candidate $c_{j}^{i}$ for the chunk *i* and travel forward to the vertex $v_{0}^{i+1}$, or else, it will continue exploring and attempt to travel to the vertex $v_{j+1}^{i}$. Otherwise, it will travel backwards to a vertex from the previous chunk $v_{k}^{i-1},0\leq k<m_{i-1}$, when there is no suitable chunk candidate for the current chunk *i*.

As can be noted, this variant uses a simple backtracking strategy. In order to speed up the pruning process, we will make use of two precomputed tables minArray(maxArray). The entry minArray[i](maxArray[i]) holds the global minimum (maximum) value that can be reached from chunk *i* to chunk N−1. In other words,

$$\mathrm{minArray}\left[i\right]=\min\left\{\sum_{j=i}^{N-1}c_{k_{j}}^{j}.score:c_{k_{j}}^{j}\in L^{j}\right\},0\leq i<N,$$

$$\mathrm{maxArray}\left[i\right]=\max\left\{\sum_{j=i}^{N-1}c_{k_{j}}^{j}.score:c_{k_{j}}^{j}\in L^{j}\right\},0\leq i<N,$$

with minArray[N]=maxArray[N]=0.

Additionally, note that when each list of chunk candidates $L^{i}=\left[c_{0}^{i},c_{1}^{i},\ldots ,c_{m_{i}-1}^{i}\right],0\leq i<N$, is in decreasing order based on the score component of its chunk candidates, we can compute the entry minArray(maxArray) by computing

$$\mathrm{maxArray}\left[i\right]=\sum_{j=i}^{N-1}c_{0}^{j}.score$$

and

$$\mathrm{minArray}\left[i\right]=\sum_{j=i}^{N-1}c_{m_{j}-1}^{j}.score$$

Therefore, the basic variant is sped up by computing maxS (minS), which is the maximum(minimum) score that can be obtained from the current chunk candidate, and then by checking if the intersection of the intervals [minS,maxS] and $\left[B_{1},B_{2}\right]$ is not empty.

#### 4.5.1. Setup

We now introduce a couple of tools that we will use to describe the algorithm, using the following notations. S will denote a stack. This data structure supports two basic methods [30]. Firstly, the method S.pop() removes the element at the top of this stack and returns that element as the value of this function. Secondly, the method S.push(e) pushes *e* onto the top of this stack. This stack *S* will store 4-tuples of the form (score,i,j,indices), where score is the accumulated score at any stage of the algorithm, *i* and *j* are the indices for the chunk candidate $c_{j}^{i}$, and indices is an array of positive integers holding the indices of the selected chunk candidates, i.e., the chunk candidate $c_{indices[k]}^{k}$ is assigned to chunk *k* and for each k, 0≤k≤i.

#### 4.5.2. Complete Algorithm

Firstly, at the initialisation stage, the 4-tuple (0,0,0,[]) will be inserted into the stack S. The main loop of this algorithm will call the function $\mathrm{nextCandidate}(S,B_{1},B_{2})$, defined in Algorithm 8, as long as the stack S is not empty. Specifically the main loop will call this function to obtain a key candidate of which its score is in the range $\left[B_{1},B_{2}\right]$. Algorithm 8 will then attempt to find such a candidate, and once it has found such a candidate, it will return the candidate to the main loop (at this point, S may not be empty). The main loop will get the key candidate, process or test it, and continue calling the function $\mathrm{nextCandidate}(S,B_{1},B_{2})$ as long as S is not empty. Because of the use of the stack S, the state of Algorithm 8 will not be lost; therefore, each time the main loop calls it, it will return a new key candidate of which its score lies in the interval $\left[B_{1},B_{2}\right]$. The main loop will terminate once all possible key candidates of which their scores are within the interval $\left[B_{1},B_{2}\right]$ have already been generated, which will happen once the stack is empty.

**Algorithm 8** outputs a key candidate in the interval $\left[B_{1},B_{2}\right]$.
1:**function**NextCandidate($S,B_{1},B_{2}$)2:    **while** S is not empty **do**3:        (aScore,i,j,indices)←S.pop();4:        **if** $j<L^{i}.\mathrm{size}\left(\right)-1$ **then**5:           S.push((aScore,i,j+1,indices));6:        **end if**7:        $uScore\leftarrow aScore+c_{j}^{i}.score$;8:        maxS←uScore+maxArray[i+1];9:        minS←uScore+minArray[i+1];10:        **if** $\mathrm{maxS}\geq B_{1}$ **and**  $\mathrm{minS}\leq B_{2}$ **then**11:           **if** $uScore\leq B_{2}$ **then**12:               **if** i=N−1 **then**13:                   **if** $B_{1}\leq uScore$ **then**14:                       indices←indices ‖ [j];15:                       $c\leftarrow \mathrm{combine}(c_{indices[0]}^{0},\ldots ,c_{indices[N-1]}^{N-1});$16:                       break;17:                   **end if**18:               **else**19:                   S.push((aScore,i+1,0,indices ‖ [j]));20:               **end if**21:           **end if**22:        **end if**23:    **end while**24:    **return** c;25:
**end function**



#### 4.5.3. Memory Consumption

We claim that, at any stage of the algorithm, there are at most N 4-tuples stored in the stack S. Indeed, after the stack is initialised, it only contains the 4-tuple (0,0,0,[]). Note that, during the execution of a **while** iteration, a 4-tuple is removed out of the stack and two new 4-tuples might be inserted. Hence, after *s* **while** iterations have been completed, there will be $N_{S}^{s}=1+\left(-1+l_{1}\right)+\left(-1+l_{2}\right)+\left(-1+l_{3}\right)+\left(-1+l_{4}\right)+\cdots +\left(-1+l_{s}\right)$ 4-tuples, where $0\leq l_{r}\leq 2$, for 1≤r≤s.

Suppose now that the algorithm is about to execute the $k_{th}$ **while** iteration during which the first valid key candidate will be returned. Therefore, $N_{S}^{k-1}=1+\left(-1+l_{1}\right)+\left(-1+l_{2}\right)+\left(-1+l_{3}\right)+\left(-1+l_{4}\right)+\cdots +\left(-1+l_{k-1}\right)\leq N.$ During the execution of the $k_{th}$ **while** iteration, a 4-tuple will be removed and only a new 4-tuple will be considered for insertion in the stack. Therefore, we have that $N_{S}^{k}=N_{S}^{k-1}-1+l_{k}\leq N-1+l_{k}\leq N$, since $0\leq l_{k}\leq 1$. Applying a similar reasoning, we have $N_{S}^{n}\leq N$ for n>k.

#### 4.5.4. Parallelization

One of the most interesting features of the previous algorithm is that it is parallelizable. The original authors suggested as a parallelization method to run instances of the algorithm over different disjoint intervals [16]. Although this method is effective and has a potential advantage as the different instances will produce nonoverlapping lists of key candidates with the instance searching over the first interval producing the most-likely key candidates, it is not efficient since each instance will inevitably repeat a lot of the work done by the other instances. Here, we propose another parallelization method that partitions the search space to avoid the repetition of work.

Suppose that we want to have *t* parallel, independent tasks $T_{1},T_{2},T_{3},\ldots ,T_{t}$ to search over a given interval in parallel. Let $L^{i}=\left[c_{0}^{i},c_{1}^{i},\ldots ,c_{m_{i}-1}^{i}\right]$ be the list of chunk candidates for chunk *i*, 0≤i≤N−1.

We first assume that $t\leq m_{0}$, where $m_{0}$ is the size of $L^{0}$. In order to construct these tasks, we partition $L^{0}$ into *t* disjoint, roughly equal-sized sublists $L_{j}^{0},1\leq j\leq t$. We set each task $T_{j}$ to perform its enumeration over the given interval but only consider the lists of chunk candidates $L_{j}^{0},L^{1},\ldots ,L^{N-1}$.

Note that the previous startegy can be easily generalised for $m_{0}<t\ll \prod_{k=0}^{N-1}m_{k}$. Indeed, first, find the smallest integer *l*, with 0<l<N−1, such that $\prod_{k=0}^{l-1}m_{k}<t\leq \prod_{k=0}^{l}m_{k}$. We then construct the list of chunk candidates $L^{0,\ldots ,l}$ as follows. For each (l+1)-tuple $\left(c_{j_{0}}^{0},c_{j_{1}}^{1},\ldots ,c_{j_{l}}^{l}\right)$, with $c_{j_{k}}^{k}\in L^{k},0\leq j_{k}<m_{k},0\leq k\leq l$, the chunk candidate $c^{j_{0},j_{1},\ldots ,j_{l}}$ is constructed by calculating $c^{j_{0},j_{1},\ldots ,j_{l}}.score=\sum_{k=0}^{l}c_{j_{k}}^{k}.score$ and by setting $c^{j_{0},j_{1},\ldots ,j_{l}}.value=\left[c_{j_{0}}^{0}.value,\ldots ,c_{j_{l}}^{l}.value\right]$, and then, $c^{j_{0},j_{1},\ldots ,j_{l}}$ is added to $L^{0,\ldots ,l}$. We then partition $L^{0,\ldots ,l}$ into *t* disjoint, roughly equal-sized sublists $L_{j}^{0,\ldots ,l},1\leq j\leq t$ and finally set each task $T_{j}$ to perform its enumeration over the given interval but only consider the lists of chunk candidates $L_{j}^{0,\ldots ,l},L^{l+1},\ldots ,L^{N-1}$. Note that the workload assigned to each enumerating task is a consequence of the selected method for partitioning the list $L^{0,\ldots ,l}$.

Additionally, both parallelization methods can be combined by partitioning the given interval $\left[B_{1},B_{2}\right]$ into $n_{s}$ disjoint subintervals and by searching each such subinterval with $t_{k}$ tasks, hence amounting to $\sum_{k=1}^{n_{s}}t_{k}$ enumerating tasks.

### 4.6. Threshold Algorithm

Algorithm 8 shares some similarities with the algorithm Threshold introduced in the research paper [14], since Threshold also makes use of an array (partialSum) similar to the array minArray to speed up the pruning process. However, Threshold works with nonnegative integer values (weights) rather than scores. Threshold restricts the scores to weights such that the smallest weight is the likeliest score by making use of a function that converts scores into weights [14].

Assuming the scores have already been converted to weights, Threshold first sorts each list of chunk candidates $L^{i}=\left[c_{0}^{i},c_{1}^{i},\ldots ,c_{m_{i}-1}^{i}\right],0\leq i<N$ in ascending order based on the score/weight component of its chunk candidates. It then computes the entries of partialSum by first setting partialSum[N−1]=0 and then by computing

$$\mathrm{partialSum}\left[i\right]=\mathrm{partialSum}\left[i+1\right]+c_{0}^{i}.score\;for\;i=N-2,N-3,\ldots ,0$$

Threshold then enumerates all the key candidates of which their accumulated total weight lies in a range of the form $\left[0,W_{t}\right)$, where $W_{t}$ is a parameter. To do so, it performs a similar process to Algorithm 8 by using its precomputed table (partialSum) to avoid useless paths, hence improving the pruning process. This enumeration process performed by Threshold is described in Algorithm 9.

According to its designers, this algorithm may perform a nonoptimal enumeration to a depth of $2^{40}$ if some adjustments are made in the data structure *L* used to store the key candidates. However, its primary drawback is that it must always start enumerating from the most likely key. Consequently, whilst the simplicity and relatively strong time complexity of Threshold is desirable, in a parallelized environment, it can only serve as the first enumeration algorithm (or can only be used in the first search task). Threshold, therefore, was not implemented and, hence, is not included in the comparison made in Section 5.

**Algorithm 9** enumerates all key candidate in the interval $\left[0,W_{t}\right]$.
1:**function**threshold($i,w,K,W_{t},L$)2:    **for** $j=0\;to\;m_{i}$ **do**3:        $newW\leftarrow w+c_{j}^{i}.score;$4:        **if** $\left(newW+\mathrm{partialSum}\left[i\right]\right)>W_{t})$ **then**5:           break;6:        **else**7:           **if** i=N−1 **then**8:               $K_{i}\leftarrow j;$9:               $c\leftarrow \mathrm{combine}(c_{K[0]}^{0},c_{K[1]}^{1},\ldots ,c_{K[N-1]}^{N-1});$10:               L←L.add(c);11:           **else**12:               $K_{i}\leftarrow j;$13:               $L\leftarrow \mathrm{threshold}(i+1,newW,K,W_{t},L);$14:           **end if**15:        **end if**16:    **end for**17:    **return** *L*;18:
**end function**



### 4.7. A Weight-Based Key Enumeration Algorithm

In this subsection, we will describe a nonoptimal enumeration algorithm based on the algorithm introduced in the research paper [12]. This algorithm differs from the original algorithm in the manner in which this algorithm builds a precomputed table (iRange) and uses it during execution to construct key candidates of which their total accumulated score is equal to a certain accumulated score. This algorithm shares similarities with the stack-based, depth-first key enumeration algorithm described in Section 4.5 because both algorithms essentially perform a depth-first search in the undirected graph *G*. However, this algorithm controls pruning by the accumulated total score that a key candidate must reach to be accepted. To achieve this, the scores are restricted to positive integer values (weights), which may be derived from a correlation value in a side-channel analysis attack.

This algorithm starts off by generating all key candidates with the largest possible accumulated total weight $W_{1}$ and then proceeds to generate all key candidates of which their accumulated total weight are equal to the second largest possible accumulated total weight $W_{2}$, and so forth, until it generates all key candidates with the minimum possible accumulated total weight $W_{N}$. To find a key candidate with a weight equal to a certain accumulated weight, this algorithm makes use of a simple backtracking strategy, which is efficient because impossible paths can be pruned early. The pruning is controlled by the accumulated weight that must be reached for the solution to be accepted. To achieve a fast decision process during backtracking, this algorithm precomputes tables for minimal and maximal accumulated total weights that can be reached by completing a path to the right, like the tables minArray and maxArray introduced in Section 4.5. Additionally, this algorithm precomputes an additional table, iRange.

Given 0≤i≤N and minArray[i]≤w≤maxArray[i], the entry iRange[i][w] points to a list of integers $L^{\left(i,w\right)}=\left[k_{0}^{\left(i,w\right)},k_{1}^{\left(i,w\right)},\ldots ,k_{n}^{\left(i,w\right)}\right]$, where each entry represents a distinct index of the list $L^{i}$, i.e., $0\leq k_{j}^{\left(i,w\right)}\neq k_{l}^{\left(i,w\right)}<m_{i}$ for j≠l. The algorithm uses these indices to construct a chunk candidate with an accumulated score *w* from chunk *i* to chunk N−1.

In order to compute this table, we use the observation that for a given entry $k_{j}^{\left(i,w\right)}$ of iRange[i][w], the list iRange[i+1][cw], with $cw=w-c_{k_{j}^{\left(i,w\right)}}^{i}.score$, must be defined and be nonempty. So we first set the entry iRange[N][0] to [0] and then proceed to compute the entries for i=N−1,…,0 and w=minArray[i],…,maxArray[i]. Algorithm 10 describes precisely how this table is precomputed.

**Algorithm 10** precomputes the table iRange.
1:**function**PrecomputeIRange( )2:    iRange[N][0]←[0];3:    **for** i=N−1 to 0 **do**4:        **for** w=minArray[i] to maxArray[i] **do**5:           $L^{\left(i,w\right)}\leftarrow \left[\right];$6:           **for** k=0 to $m_{i}-1$ **do**7:               $cw\leftarrow w-c_{k}^{i}.score$;8:               **if** iRange[i+1][cw].size()>0 **then**9:                   $L^{\left(i,w\right)}.\mathrm{add}\left(k\right)$;10:               **end if**11:           **end for**12:           **if** $L^{\left(i,w\right)}.\mathrm{size}\left(\right)>0$ **then**13:               $\mathrm{IRange}\left[i\right]\left[w\right]\leftarrow L^{\left(i,w\right)}$;14:           **end if**15:        **end for**16:    **end for**17:    **return** IRange18:
**end function**



#### 4.7.1. Complete Algorithm

Algorithm 11 describes the backtracking strategy more precisely, making use of the precomputed tables for pruning impossible paths. The integer array TWeights contains accumulated weights in a selected order, where an entry w∈TWeights must satisfy that the list iRange[0][w] is non-empty, i.e., iRange[0][w].size()>0. This helps in constructing a key candidate with an accumulated score *w* from chunk 0 to chunk N−1. In particular, TWeights may be set to $\left[W_{1},W_{2},\ldots ,W_{N}\right]$, i.e., the array containing all possible accumulated scores that can be reached from chunk 0 to chunk N−1.

Furthermore, the order in which the elements in the array TWeights are arranged is important. For this array $\left[W_{1},W_{2},\ldots ,W_{N}\right]$, for example, the algorithm will first enumerate all key candidates with accumulated weight $W_{1}$ and then all those with accumulated weight $W_{2}$ and so on. This guarantees a certain quality, since good key candidates will be enumerated earlier than worse ones. However, key candidates with the same accumulated weight will be generated in no particular order, so a lack of precision in converting scores to weights will lead to some decrease of quality.

**Algorithm 11** enumerates key candidates for given weights.
1:**function**KeyEnumeration(TWeights, iRange)2:    **for** w∈TWeights **do**3:        i←0;4:        k[0]←(0,iRange[0][w].get(0)); 2-tuple $\left(e_{1},e_{2}\right)$5:        cw←w;6:        **while** i≥0 **do**7:           **while** i<N−1 **do**8:               $cw\leftarrow cw-c_{k\left[i\right].e_{2}}^{i}.score;$9:               i←i+1;10:               k[i]←(0,iRange[i][cw].get(0));11:           **end while**12:           $c\leftarrow \mathrm{combine}(c_{k\left[0\right].e_{2}}^{0},c_{k\left[1\right].e_{2}}^{1},\ldots ,c_{k\left[N-1\right].e_{2}}^{N-1});$13:           Test(c);14:           **while** i≥0 **and** $k\left[i\right].e_{1}\geq \left(\mathrm{iRange}\left[i\right]\left[cw\right].\mathrm{size}\left(\right)-1\right)$ **do**15:               i←i−1;16:               **if** i≥0 **then**17:                   $cw\leftarrow cw+c_{k\left[i\right].e_{2}}^{i}.score;$18:               **end if**19:           **end while**20:           **if** i≥0 **then**21:               $k\left[i\right]\leftarrow (k\left[i\right].e_{1}+1,\mathrm{iRange}\left[i\right]\left[cw\right].\mathrm{get}\left(k\left[i\right].e_{1}+1\right));$22:           **end if**23:        **end while**24:    **end for**25:
**end function**



Algorithm 11 makes use of the table k with N entries, each of which is a 2-tuple of the form $\left(e_{1},e_{2}\right)$ with $e_{1}$ and $e_{2}$ integers. For a given tuple k[i], the component $k\left[i\right].e_{1}$ is an index of some list iRange[i][w], with minArray[i]≤w≤maxArray[i], while $k\left[i\right].e_{2}$ is the corresponding value, i.e., $k\left[i\right].e_{2}=\mathrm{iRange}\left[i\right]\left[w\right].\mathrm{get}\left(k\left[i\right].e_{1}\right)$. The value of $k\left[i\right].e_{1}$ allows the algorithm to control if the list iRange[i][w] has been traveled completely or not, while the second component allows the algorithm to retrieve the chunk candidate of index $k\left[i\right].e_{2}$ of $L^{i}$. This is done to avoid recalculating $k\left[i\right].e_{2}$ each time it is required during the execution of the algorithm.

We will now analyse Algorithm 11. Suppose that w∈TWeights; hence, iRange[0][w].size()>0. The algorithm will then set k[0] to $\left(0,e_{2}^{\left(0\right)}\right)$, with $e_{2}^{\left(0\right)}$ being the integer from the entry of index 0 of iRange[0][w], and then set cw to *w* (lines 3 to 5). We claim that the main while loop (lines 6 to 23) at each iteration will compute k[i] for 0≤i≤N−1 such that the key candidate c constructed at line 12 will have an accumulated score *w*.

Let us set $cw_{0}=w$. We know that the list $\mathrm{iRange}\left[0\right]\left[cw_{0}\right]$ is non-empty; hence, for any entry $e_{2}^{\left(0\right)}$ in the list $\mathrm{iRange}\left[0\right]\left[cw_{0}\right]$, the list $\mathrm{iRange}\left[1\right]\left[cw_{1}\right]$ is non-empty, where

$$\mathrm{minArray}\left[1\right]\leq cw_{1}=cw_{0}-c_{e_{2}^{\left(0\right)}}^{0}.score\leq \mathrm{maxArray}\left[1\right].$$

Likewise, for any entry $e_{2}^{\left(1\right)}$ in the list $\mathrm{iRange}\left[1\right]\left[cw_{1}\right]$, the list $\mathrm{iRange}\left[2\right]\left[cw_{2}\right]$ is non-empty, where

$$\mathrm{minArray}\left[2\right]\leq cw_{2}=cw_{1}-c_{e_{2}^{\left(1\right)}}^{1}.score\leq \mathrm{maxArray}\left[2\right].$$

Hence, for 0≤i<N, we have that, for any given entry $e_{2}^{\left(i\right)}$ in the list $\mathrm{iRange}\left[i\right]\left[cw_{i}\right]$, the list $\mathrm{iRange}\left[i+1\right]\left[cw_{i+1}\right]$ is non-empty, where

$$\mathrm{minArray}\left[i+1\right]\leq cw_{i+1}=cw_{i}-c_{e_{2}^{\left(i\right)}}^{i}.score\leq \mathrm{maxArray}\left[i+1\right].$$

Note that, when i=N−1, the list iRange[i+1][0]=[0] is non-empty and $cw_{i+1}=0$.

Given k[0],k[1],…,k[j] are already set for some 0≤j<N−1; the first inner **while** loop (lines 7 to 11) will set $k\left[i\right]=\left(0,e_{2}^{\left(i\right)}\right)$, where $e_{2}^{\left(i\right)}$ holds the entry of index 0 of $\mathrm{iRange}\left[i\right]\left[cw_{i}\right]$, for 0<j<i≤N−1. Therefore, once the **while** loop ends, i=N−1 and $cw_{i+1}=cw_{N}=cw_{i}-c_{e_{2}^{\left(i\right)}}^{i}.score=0$. Hence, the key candidate constructed from the second components $k\left[l\right].e_{2},0\leq l\leq N-1,$ will have an accumulated score *w*. In particular, the first time k[0] is set, and so, the first inner **while** loop will calculate k[1],…,k[N−1].

Since there may be more than one key candidate with an accumulated score *w*, the second inner **while** loop (lines 14 to 19) will backtrack to a chunk 0≤i<N, from which a new key candidate with accumulated score *w* can be constructed. This is done by simply moving backwards (line 15) and updating $cw_{i+1}$ to $cw_{i}=cw_{i+1}+c_{k[i].e2}^{i}.score$ until there is an *i*, 0≤i<N, such that $k\left[i\right].e_{1}<\mathrm{iRange}\left[i\right]\left[cw_{i}\right].\mathrm{size}\left(\right)-1$.

1. If there is such an *i*, then the instruction at line 21 will update k[i] to $\left(k\left[i\right].e_{1}+1,\mathrm{iRange}\left[i\right]\left[cw_{i}\right].\mathrm{get}\left(k\left[i\right].e_{1}+1\right)\right)$. This means that the updated value for the second component of k[i] will be a valid index in $L^{i}$, so $c_{k[i].e2}^{i}$ will be the new chunk candidate for chunk *i*. Then, the first inner **while** loop (lines 7 to 11) will again execute and compute the indices for the remaining chunk candidates in the lists $L^{i+1},\ldots ,L^{N-1}$ such that the resulting key candidate will have the accumulated score *w*.
2. Otherwise, if i<0, then the main **while** loop (lines 6 to 23) will end and *w* will be set to a new value from TWeights, since all key candidates with an accumulated score *w* have just been enumerated.

#### 4.7.2. Parallelization

Suppose we would like to have *t* tasks $T_{1},T_{2},T_{3},\cdots ,T_{t}$ executed in parallel to enumerate key candidates of which the accumulated total weights are equal to those in the array TWeights. We can split the array TWeights into *t* disjoint sub-arrays ${\mathrm{TWeights}}_{i}$ and then set each task $T_{i}$ to run Algorithm 11 through the sub-array ${\mathrm{TWeights}}_{i}$. As an example of a partition algorithm to distribute the workload among the tasks, we set the sub-array ${\mathrm{TWeights}}_{i}$ to contain elements with indices congruent to *i* mod *t* from TWeights. Additionally, note that, if we have access to the number of candidates to be enumerated for each score in the array TWeights beforehand, we may design a partition algorithm for distributing the workload among the tasks almost evenly.

#### 4.7.3. Run Times

We assume each list of chunk candidates $L^{i}=\left[c_{0}^{i},c_{1}^{i},\ldots ,c_{m_{i}-1}^{i}\right],0\leq i<N$, is in decreasing order based on the score component of its chunk candidates. Regarding the run time for computing the tables maxArray and minArray, note that each entry of the table minArray(maxArray) can be computed as explained in Section 4.5. Therefore, the run time of such an algorithm is Θ(N).

Regarding the run time for computing iRange, we will analyse Algorithm 10. This algorithm is composed of three **For** blocks. For each *i*, 0≤i<N, the **For** loop from line 4 to line 15 will be executed $r_{i}$ times, where $r_{i}=\mathrm{maxArray}\left[i\right]-\mathrm{minArray}\left[i\right]+1$. For each iteration, the innermost **For** block (lines 6 to 11) will execute simple instructions $m_{i}$ times. Therefore, once the innermost block has finished, its run time will be $T_{3}\cdot m_{i}+C_{3}$, where $T_{3}$ and $C_{3}$ are constants. Then, the **if** block (lines 12 to 14) will be attempted and its run time will be $C_{2}$, where $C_{2}$ is another constant. Therefore, the run time for an iteration of the **For** loop (lines 4 to 15) will be $T_{3}\cdot m_{i}+C_{2}+C_{3}$. Therefore, the run time of Algorithm 10 is $\sum_{i=0}^{N-1}r_{i}\left(T_{3}\cdot m_{i}+C_{2}+C_{3}\right)$. More specifically,

$$\sum_{i=0}^{N-1}\left(\mathrm{maxArray}\left[i\right]-\mathrm{minArray}\left[i\right]+1\right)\left(T_{3}\cdot m_{i}+C_{2}+C_{3}\right).$$

As noted, this run time depends heavily on $r_{i}=\mathrm{maxArray}\left[i\right]-\mathrm{minArray}\left[i\right]+1$. Now, the size of the range [minArray[i],maxArray[i]] relies on the scaling technique used to get a positive integer from a real number. The more accurate the scaling technique is, the more different integer scores there will be. Hence, if we use an accurate scaling technique, we will probably get larger $r_{i}$.

We will analyse the run time for Algorithm 11 to generate all key candidates of which their total accumulated weight is *w*. Let us assume there are $N_{w}$ key candidates of which their total accumulated score is equal to *w*.

First, the run time for instructions at lines 3 to 5 is constant. Therefore, we will only focus on the **while** loop (lines 6 to 23). In any iteration, the first inner **while** loop (lines 7 to 11) will execute and compute the indices for the remaining chunk candidates in the lists $L^{i},\ldots ,L^{N-1}$, with *i* starting at any number in [0,N−2], such that the resulting key candidate will have the accumulated score *w*. Therefore, its run time is at most C·(N−1), where *C* is a constant, i.e., it is O(N). The instruction at line 12 will combine all chunks from 0 to N−1, and hence, its run time is also O(N). The next instruction Test(c) will test c, and its run time will depend on the scenario in which the algorithm is being run. Let us assume its run time is O(T(N)), where *T* is a function.

Regarding the second inner **while** loop (lines 14 to 19), this loop will backtrack to a chunk *i* with 0≤i<N, from which a new key candidate with accumulated score *w* can be constructed. This is done by simply moving backwards while computing some simple operations. Therefore, the run time for the second inner **while** loop is at most D·(N−1), where *D* is a constant, i.e., it is O(N). Therefore, the run time for generating all key candidates of which the total accumulated score is *w* will be $O(N_{w}\cdot \left(N+T\left(N\right)\right))$.

#### 4.7.4. Memory Consumption

Besides the precomputed tables, it is easy to see that Algorithm 11 makes use of negligible memory while enumerating key candidates. Indeed, testing key candidates is done on the fly to avoid storing them during enumeration. However, the table iRange may have many entries.

Let $N_{e}$ be the number of entries of the table iRange. Line 2 of Algorithm 10 will create the entry iRange[N][0] that points to the list [0]. Hence, after the instruction at line 2 has been executed, $N_{e}=1$. Let us consider the **For** block from line 4 to line 15. For each *i*, 0≤i<N, let $W_{i}$ be the set of different values *w* in the range [minArray[i],maxArray[i]] such that $L^{\left(i,w\right)}$ is non-empty. After the iteration for *i* has been executed, the table iRange will have $|W_{i}|$ new entries, each of which will point to a non-empty list, with $0<|W_{i}|\leq r_{i}.$ Therefore, $N_{e}=1+\sum_{i=0}^{N-1}\left|W_{i}\right|$ after Algorithm 10 has completed its execution.

Note that $|W_{i}|$ may increase if the range [minArray[i],maxArray[i]] is large. The size of this interval relies on the scaling technique used to get a positive integer from a real number. The more accurate the scaling technique is, the more different integer scores there will be. Hence, if we use an accurate scaling technique, we will probably get larger $r_{i}$, making it likely for $|W_{i}|$ to increase. Therefore, the table iRange may have many entries.

Regarding the number of bits used in memory to store the table iRange, let us suppose that an integer is stored in $B_{int}$ bits and that a pointer is stored in $B_{p}$ bits. Once Algorithm 10 has completed its execution, we know that iRange[i][w] will point to the list $L^{\left(i,w\right)}$, with 0≤i≤N and $w\in W_{i}.$ Moreover, by definition, we know that the list $L^{\left(N,0\right)}$ will be the list [0], while any other list $L^{\left(i,w\right)}$, 0≤i<N and $w\in W_{i}$, will have $n^{\left(i,w\right)}$ entries, with $1\leq n^{\left(i,w\right)}\leq m_{i}$. Therefore, the number of bits iRange occupies in memory after Algorithm 11 has completed its execution is

$$T_{b}=B_{int}+B_{p}+\sum_{i=0}^{N-1}\sum_{w\in W_{i}}\left(n^{\left(i,w\right)}\cdot B_{int}+B_{p}\right).$$

Since $1\leq n^{\left(i,w\right)}\leq m_{i}$, we have

$$B_{int}+B_{p}+\sum_{i=0}^{N-1}|W_{i}|\cdot \left(B_{int}+B_{p}\right)\leq T_{b}\leq B_{int}+B_{p}+\sum_{i=0}^{N-1}\left|W_{i}\right|\cdot \left(m_{i}\cdot B_{int}+B_{p}\right).$$

### 4.8. A Key Enumeration Algorithm using Histograms

In this subsection, we will describe a nonoptimal key enumeration algorithm introduced in the research paper [17].

#### 4.8.1. Setup

We now introduce a couple of tools that we will use to describe the sub-algorithms used in the algorithm of the research paper [17], using the following notations: *H* will denote a histogram, $N_{b}$ will denote a number of bins, *b* will denote a bin, and *x* will denote a bin index.

##### Linear Histograms

The function $H_{i}=\mathrm{createHist}\left(L^{i},N_{b}\right)$ creates a standard histogram from the list of chunk candidates $L_{i}$ with $N_{b}$ linearly spaced bins.

Given a list of chunk candidates $L^{i}$, the function createHist will first calculate both the minimum score min and maximum score max among all the chunk candidates in $L^{i}$. It will then partition the interval I=[min,max] into subintervals $I_{0}=\left[min,min+\sigma \right),I_{1}=\left[min+\sigma ,min+2\sigma \right),\ldots ,I_{N_{b}-1}=\left[min+\left(N_{b}-1\right)\sigma ,max\right]$, where $\sigma =\frac{max-min}{N_{b}}.$ It then will proceed to build the list $L_{H_{i}}$ of size $N_{b}$. The entry $0\leq x<N_{b}$ of $L_{H_{i}}$ will point to a list that contains all chunk candidates from $L^{i}$ such that their scores lie in $I_{x}$. The returned standard histogram $H_{i}$ is therefore stored as the list $L_{H_{i}}$ of which its entries will point to lists of chunk candidates. For a given bin index *x*, $L_{H_{i}}.\mathrm{get}\left(x\right)$ outputs the list of chunk candidates contained in the bin of index *x* of $H_{i}.$ Therefore, $H_{i}\left[x\right]=L_{H_{i}}.\mathrm{get}\left(x\right).\mathrm{size}\left(\right)$ is the number of chunk candidates in the bin of index *x* of $H_{i}$. The run time for $\mathrm{createHist}(L^{i},N_{b})$ is $\Theta (m_{i}+N_{b})$.

##### Convolution

This is the usual convolution algorithm which computes $H_{1:2}=\mathrm{conv}\left(H_{1},H_{2}\right)$ from two histograms $H_{1}$ and $H_{2}$ of sizes $n_{1}$ and $n_{2}$, respectively, where $H_{1:2}\left[k\right]=\sum_{i=0}^{k}H_{1}\left[i\right]\cdot H_{2}\left[k-i\right].$ The computation of $H_{1:2}$ is done efficiently by using Fast Fourier Transformation (FFT) for polynomial multiplication. Indeed, the array $\left[H_{j}\left[0\right],H_{j}\left[1\right],\ldots ,H_{j}\left[n_{j}-1\right]\right]$ is seen as the coefficient representation of $P_{j}=H_{j}\left[0\right]+H_{j}\left[1\right]x+\ldots +H_{j}\left[n_{j}-1\right]x^{n_{j}-1}$ for j=1,2. In order to get $H_{1:2}$, we multiply the two polynomials of degree-bound $n=\max(n_{1},n_{2})$ in time Θ(nlogn), with both the input and output representations in coefficient form [30]. The convoluted histogram $H_{1:2}$ is therefore stored as a list of integers.

##### Getting the Size of a Histogram

The method size() returns the number of bins of a histogram. This method simply returns L.size(), where *L* is the underlying list used to represent the histogram.

##### Getting Chunk Candidates from a Bin

Given a standard histogram $H_{i}$ and an index $0\leq x<H_{i}.\mathrm{size}\left(\right)$, the method $H_{i}.\mathrm{get}\left(x\right)$ outputs the list of all chunk candidates contained in the bin of index *x* of $H_{i}$, i.e., this method simply returns the list $L_{H_{i}}.\mathrm{get}\left(x\right).$

#### 4.8.2. Complete Algorithm

This key enumeration algorithm uses histograms to represent scores, and the first step of the key enumeration is a convolution of histograms modelling the distribution of the N lists of scores. This step is detailed in Algorithm 12.

**Algorithm 12** computes standard and convoluted histograms.
1:**function**createHistograms($L^{0},L^{1},\ldots ,L^{N-1},N_{b}$)2:    $H_{0}\leftarrow \mathrm{createHist}\left(L^{0},N_{b}\right)$;3:    $H_{1}\leftarrow \mathrm{createHist}\left(L^{1},N_{b}\right)$;4:    $H_{0:1}\leftarrow \mathrm{conv}\left(H_{0},H_{1}\right)$;5:    **for** i=2 to N−1 **do**6:        $H_{i}\leftarrow \mathrm{createHist}\left(L^{i},N_{b}\right)$;7:        $H_{0:i}\leftarrow \mathrm{conv}\left(H_{i},H_{0:i-1}\right)$;8:    **end for**9:    **return** $H=[H_{0},H_{1},\ldots ,H_{N-1},H_{0:1},\ldots ,H_{0:N-1}]$;10:
**end function**



Based on this first step, this key enumeration algorithm allows enumerating key candidates that are ranked between two bounds $R_{1}$ and $R_{2}$. In order to enumerate all keys ranked between the bounds $R_{1}$ and $R_{2}$, the corresponding indices of bins of $H_{0:N-1}$ have to be computed, as described in Algorithm 13. It simply sums the number of key candidates contained in the bins starting from the bin containing the highest scoring key candidates until we exceed $R_{1}$ and $R_{2}$ and returns the corresponding indices $x_{start}$ and $x_{stop}.$

**Algorithm 13** computes the indices’ bounds.
1:**function**computeBounds($R_{1},R_{2}$)2:    $start\leftarrow H_{0:N-1}.\mathrm{size}\left(\right)$;3:    $cnt_{start}\leftarrow 0$;4:    **while** $cnt_{start}<R_{1}$ **do**5:        start←start−1;6:        $cnt_{start}\leftarrow cnt_{start}+H_{0:N-1}\left[start\right]$;7:    **end while**8:    $x_{start}\leftarrow start$;9:    **while** $cnt_{start}<R_{2}$ **do**10:        start←start−1;11:        $cnt_{start}\leftarrow cnt_{start}+H_{0:N-1}\left[start\right]$;12:    **end while**13:    $x_{stop}\leftarrow start$;14:    **return** $x_{start},x_{stop}$;15:
**end function**



Given the list of histograms of scores *H* and the indices of bins of $H_{0:N-1}$ between which we want to enumerate, the enumeration simply consists of performing a backtracking over all the bins between $x_{start}$ and $x_{stop}$. More precisely, during this phase, we recover the bins of the initial histograms (i.e., before convolution) that were used to build a bin of the convoluted histogram $H_{0:N-1}$. For a given bin *b* with index *x* of $H_{0:N-1}$, we have to run through all the non-empty bins $b_{0},\ldots ,b_{N-1}$ of indices $x_{0},\ldots ,x_{N-1}$ of $H_{0},\ldots ,H_{N-1}$ such that $x_{0}+\ldots +x_{N-1}=x$. Each $b_{i}$ will then contain at least one and at most $m_{i}$ chunk candidates of the list $L^{i}$ that we must enumerate. This leads to storing a table kf of N entries, each of which points to a list of chunk candidates. The list pointed to by the entry kf[i] holds at least one and at most $m_{i}$ chunk candidates contained in the bin $b_{i}$ of the histogram $H_{i}.$ Any combination of these N lists, i.e., picking an entry from each list, results in a key candidate.

Algorithm 14 describes more precisely this bin decomposition process. This algorithm simply follows a recursive decomposition. That is, in order to enumerate all the key candidates within a bin *b* of index *x* of $H_{0:N-1}$, it first finds two non-empty bins of indices $x_{N-1}$ and $x-x_{N-1}$ of $H_{N-1}$ and $H_{0:N-2}$, respectively. All the chunk candidates in the bin of index $x_{N-1}$ of $H_{N-1}$ will be added to the key factorisation, i.e., the entry kf[N−1] will point to the list of chunk candidates returned by $H_{N-1}.\mathrm{get}\left(x_{N-1}\right)$. It then continues the recursion with the bin of index $x-x_{N-1}$ of $H_{0:N-2}$ by finding two non-empty bins of indices $x_{N-2}$ and $x-x_{N-1}-x_{N-2}$ of $H_{0:N-2}$ and $H_{0:N-3}$, respectively, and by adding all the chunk candidates in the bin of index $x_{N-2}$ of $H_{N-2}$ to the key factorisation, i.e., kf[N−2] will now point to the list of chunk candidates returned by $H_{N-2}.\mathrm{get}\left(x_{N-2}\right)$ and so forth. Eventually, each time a factorisation is completed, Algorithm 14 calls the function processKF, which takes as input the table kf. The function processKF, as defined in Algorithm 15, will compute the key candidates from kf. This algorithm basically generates all the possible combinations from the N lists kf[i]. Note that this algorithm may be seen as a particular case of Algorithm 11. Finally, the main loop of this key enumeration algorithm simply calls Algorithm 14 for all the bins of $H_{0:N-1}$, which are between the enumeration bounds $x_{start},x_{stop}$.

**Algorithm 14** performs bin decomposition.
1:**function**DecomposeBin($H,i,x_{bin},\mathrm{kf}$)2:    **if** i=1 **then**3:        $x\leftarrow H_{0}.\mathrm{size}\left(\right)-1$;4:        **while** (x≥0) **and** $\left(x+H_{1}.\mathrm{size}\left(\right)\right)\geq x_{bin}$ **do**5:           **if** $H_{0}\left[x\right]>0$ **and** $H_{1}\left[x_{bin}-x\right]>0$ **then**6:               $\mathrm{kf}\left[0\right]\leftarrow H_{0}.\mathrm{get}\left(x\right)$;7:               $\mathrm{kf}\left[1\right]\leftarrow H_{1}.\mathrm{get}\left(x_{bin}-x\right)$;8:               processKF(kf);9:           **end if**10:           x←x−1;11:        **end while**12:    **else**13:        $x\leftarrow H_{i}.\mathrm{size}\left(\right)-1$;14:        **while** (x≥0) **and** $\left(x+H_{0:i-1}.size\left(\right)\right)\geq x_{bin}$ **do**15:           **if** $H_{i}\left[x\right]>0$ **and** $H_{0:i-1}\left[x_{bin}-x\right]>0$ **then**16:               $\mathrm{kf}\left[i\right]\leftarrow H_{i}.get\left(x\right)$;17:               $\mathrm{DecomposeBin}(H,i-1,x_{bin}-x,\mathrm{kf})$;18:           **end if**19:           x←x−1;20:        **end while**21:    **end if**22:
**end function**



#### 4.8.3. Parallelization

Suppose we would like to have *t* tasks $T_{1},T_{2},T_{3},\cdots ,T_{t}$ executing in parallel to enumerate key candidates that are ranked between two bounds $R_{1}$ and $R_{2}$ in parallel. We can then calculate the indices $x_{start}$ and $x_{stop}$ and then create the array $X=[x_{start},x_{start}-1,\ldots ,x_{stop}]$. We then partition the array X into *t* disjoint sub-arrays $X_{i}$ and finally set each task $T_{i}$ to call the function decomposeBin for all the bins of $H_{0:N-1}$ with indices in $X_{i}$.

As has been noted previously, the algorithm employed to partition the array X directly allows efficient parallel key enumeration, where the amount of computation performed by each task may be well balanced. An example of a partition algorithm that could almost evenly distribute the workload among the tasks is as follows:

1. Set *i* to 0.
2. If X is non-empty, pick an index *x* in X such that $H_{0:N-1}\left[x\right]$ is the maximum number or else return $X_{1},X_{2},\ldots ,X_{t}.$
3. Remove *x* from the array X, and add it to the array $X_{i+1}$.
4. Update *i* to (i+1)modt, and go back to Step 2.

**Algorithm 15** processes table kf.
1:**function**proccessKF(kf)2:    i←0;3:    I[i]←0;4:    **while** i≥0 **do**5:        **while** i<N−1 **do**6:           i←i+1;7:           I[i]←0;8:        **end while**9:        c←combine(kf[0].get(I[0]),…,kf[N−1].get(I[N−1]));10:        Test(c);11:        **while** i≥0 **and** I[i]≥(kf[i].size()−1) **do**12:           i←i−1;13:        **end while**14:        **if** i≥0 **then**15:           I[i]←I[i]+1;16:        **end if**17:    **end while**18:
**end function**



#### 4.8.4. Memory Consumption

Besides the precomputed histograms, which are stored as arrays in memory, it is easy to see that this algorithm makes use of negligible memory (only table kf) while enumerating key candidates. Additionally, it is important to note that each time the function processKF is called, it will need to generate all key candidates obtained by picking chunk candidates from the N lists pointed to by the entries of kf and to process all of them immediately, since the table kf may have changed. This implies that, if the processing of key candidates is left to be done after the complete enumeration has finished, each version of the table kf would need to be stored, which, again, might be problematic in terms of memory consumption.

Regarding how many bits in memory the precomputed histograms consumes, we will analyse Algorithm 12. First, note, for a given list of chunk candidates $L^{i}$ and $N_{b}$, the function $\mathrm{createHist}(L^{i},N_{b})$ will return the standard histogram $H_{i}$. This standard histogram will be stored as the list $L_{H_{i}}$ of size $N_{b}$. An entry *x* of $L_{H_{i}}$ will point to a list of chunk candidates. The total number of chunk candidates held by all the lists pointed to by the entries of $L_{H_{i}}$ is $m_{i}$. Therefore, the number of bits to store the list $L_{H_{i}}$ is $B_{p}\cdot N_{b}+B_{c}\cdot m_{i}$, where $B_{p}$ is the number of bits to store a pointer and $B_{c}$ is the number of bits to store a chunk candidate (score,[e]). The total number of bits to store all lists $L_{H_{i}},0\leq i<N,$ is

(1) $$\sum_{i=0}^{N-1}\left(B_{p}\cdot N_{b}+B_{c}\cdot m_{i}\right)=N\cdot B_{p}\cdot N_{b}+B_{c}\cdot \sum_{i=0}^{N-1}mi$$

Concerning the convoluted histograms, let us first look at $H_{0:1}=\mathrm{conv}\left(H_{0},H_{1}\right)$. We know that $H_{0:1}$ is stored as a list of integers and that these entries can be seen as the coefficients of the resulting polynomial from multiplying the polynomial $P_{0}=H_{0}\left[0\right]+H_{0}\left[1\right]x+\ldots +H_{0}\left[N_{b}-1\right]x^{N_{b}-1}$ by $P_{1}=H_{1}\left[0\right]+H_{1}\left[1\right]x+\ldots +H_{1}\left[N_{b}-1\right]x^{N_{b}-1}$. Therefore, the list of integers used to store $H_{0:1}$ has $2\cdot N_{b}-1$ entries. Following a similar reasoning to the previous one, we can conclude that the list of integers used to store $H_{0:2}=\mathrm{conv}\left(H_{2},H_{0:1}\right)$ has $3\cdot N_{b}-2$ entries. Therefore, for a given i,1≤i≤N−1, the list of integers used to store $H_{0:i}=\mathrm{conv}\left(H_{i},H_{0:i-1}\right)$ has $\left(i+1\right)\cdot N_{b}-i$ entries. The total number of entries of all the convoluted histograms $H_{0:1},H_{0:2},\ldots ,H_{0:N-1}$ is

$$\sum_{i=1}^{N-1}\left(\left(i+1\right)N_{b}-i\right)=\left(N_{b}-1\right)\cdot \frac{N\cdot (N-1)}{2}+N_{b}\cdot \left(N-1\right).$$

As expected, the total number of entries strongly depends on the values $N_{b}$ and N. If an integer is stored in $B_{int}$ bits, then the number of bits for storing all the convoluted histograms is

(2) $$B_{int}\cdot \left(N_{b}-1\right)\cdot \frac{N\cdot (N-1)}{2}+B_{int}\cdot N_{b}\cdot \left(N-1\right)$$

#### 4.8.5. Equivalence with the Path-Counting Approach

The stack-based key enumeration algorithm and the score-based key enumeration algorithm can be also used for rank computation (instead of enumerating each path, the rank version counts each path). Similarly, the histogram algorithm can also be used for rank computation by simply summing the size of the corresponding bins in $H_{0:N-1}$. These two approaches were believed to be distinct from each other. However, Martin et al. in the research paper [31] showed that both approaches are mathematically equivalent, i.e., they both compute the exact same rank when choosing their discretisation parameter correspondingly. Particularly, the authors showed that the binning process in the histogram algorithm is equivalent to the “map to weight” float-to-integer conversion used prior to their path counting algorithm (Forest) by choosing the algorithms’ discretisation parameter carefully. Additionally, in this paper, a performance comparison between their enumeration versions was carried out. The practical experiments indicated that Histogram performs best for low discretisation and that Forest wins for higher parameters.

#### 4.8.6. Variant

A recent paper by Grosso [26] introduced a variant of the previous algorithm. Basically, the author of [26] makes a small adaptation of Algorithm 14 to take into account the tree-like structure used by their rank estimation algorithm. Also, the author claims this variant has an advantage over the previous one when the memory needed to store histograms is too large.

### 4.9. A Quantum Key Search Algorithm

In this subsection, we will describe a quantum key enumeration algorithm introduced in the research paper [29] for the sake of completeness. This algorithm is constructed from a nonoptimal key enumeration algorithm, which uses the key rank algorithm given by Martin et al. in the research paper [16] to return a single key candidate (the $r_{th}$) with a weight in a particular range. We will first describe the key rank algorithm. This algorithm restricts the scores to positive integer values (weights) such that the smallest weight is the likeliest score by making use of a function that converts scores into weights [16].

Assuming the scores have already been converted to weights, the rank algorithm first constructs a matrix b with size of $N\times W_{2}$ for a given range $\left[W_{1},W_{2}\right)$ as follows. For i=N−1 and $0\leq w<W_{2}$, the entry $b_{i,w}$ contains the number of chunk candidates such that their total score plus *w* lies in the given range. Therefore, $b_{i,w}$ is given by the number of chunk candidates $c_{j}^{i},0\leq j<m_{i}$, such that $W_{1}-w\leq c_{j}^{i}.score<W_{2}-w$.

On the other hand, for i=N−2,N−3,…,0, and $0\leq w<W_{2}$, the entry $b_{i,w}$ contains the number of chunk candidates that can be constructed from the chunk *i* to the chunk N−1 such that their total score plus *w* lies in the given range. Therefore, $b_{i,w}$ may be calculated as follows. For $0\leq j<m_{i},b_{i,w}=b_{i,w}+b_{i+1,w+c_{j}^{i}.score}$ if $w+c_{j}^{i}.score<W_{2}.$

Algorithm 16 describes precisely the manner in which the matrix b is computed. Once matrix b is computed, the rank algorithm will calculate the number of key candidates in the given range by simply returning $b_{0,0}$. Note that $b_{0,0}$, by construction, contains the number of chunk candidates, with initial weight 0, that can be constructed from the chunk 0 to the chunk N−1 such that their total weight lies in the given range. Algorithm 17 describes the rank algorithm.

**Algorithm 16** creates the matrix b.
1:**function**initialise($W_{1},W_{2}$)2:    i←N−1;3:    $b\leftarrow {\left[{\left[0\right]}^{W_{2}}\right]}^{N}$;4:    **for** $w=0\;to\;W_{2}-1$ **do**5:        **for** $j=0\;to\;m_{i}-1$ **do**6:           **if** $W_{1}-w\leq c_{j}^{i}.score<W_{2}-w$ **then**7:               $b_{i,w}\leftarrow b_{i,w}+1$;8:           **end if**9:        **end for**10:    **end for**11:    **for** i=N−2to0 **do**12:        **for** $w=0\;to\;W_{2}-1$ **do**13:           **for** $j=0\;to\;m_{i}-1$ **do**14:               **if** $w+c_{j}^{i}.score<W_{2}$ **then**15:                   $b_{i,w}\leftarrow b_{i,w}+b_{i+1,w+c_{j}^{i}.score}$;16:               **end if**17:           **end for**18:        **end for**19:    **end for**20:    **return** b;21:
**end function**



**Algorithm 17** returns the number of key candidates in a given range.
1:**function**rank($W_{1},W_{2}$)2:    $b\leftarrow \mathrm{initialise}(W_{1},W_{2})$;3:    **return** $b_{0,0}$;4:
**end function**



With the help of Algorithm 17, an algorithm for requesting particular key candidates is introduced, which is described in Algorithm 18. It returns the $r_{th}$ key candidate with weight between $W_{1}$ and $W_{2}$. Note that the correctness of the function getKey follows from the correctness of b and that the algorithm is deterministic, i.e., given the same *r*, it will return the same key candidate k. Also, note that the $r_{th}$ key candidate does not have to be the $r_{th}$ most likely key candidate in the given range.

Equipped with the getkey algorithm, the authors of [29] introduced a nonoptimal key enumeration algorithm to enumerate and test all key candidates in the given range. This algorithm works by calling the function getKey to obtain a key candidate in the given range until there are no more key candidates in the given range. Also, for each obtained key candidate k, it is tested by using a testing function T returning either 1 or 0. Algorithm 19 precisely describes how this nonoptimal key enumeration algorithm works.

**Algorithm 18** returns the $r_{th}$ key candidate with weight between $W_{1}$ and $W_{2}.$
1:**function**getKey($b,W_{1},W_{2},r$)2:    **if** $r>b_{0,0}$ **then**3:        **return** ⊥;4:    **end if**    **end if**5:    $k\leftarrow {\left[0\right]}^{N}$;6:    w←0;7:    **for** i=0toN−2 **do**8:        **for** $j=0\;to\;m_{i}-1$ **do**9:           **if** $r<b_{i+1,w+c_{j}^{i}.score}$ **then**10:               $k_{i}\leftarrow j$;11:               $w\leftarrow w+c_{j}^{i}.score$;12:               **break** *j*;13:           **end if**14:           $r\leftarrow r-b_{i+1,w+c_{j}^{i}.score}$;15:        **end for**16:    **end for**17:    i←N−1;18:    **for** $j=0\;to\;m_{i}-1$ **do**19:        $v\leftarrow (W_{1}-w\leq c_{j}^{i}.score<W_{2}-w)?1:0$;20:        **if** r≤v **then**21:           $k_{i}\leftarrow j$;22:           **break** *j*;23:        **end if**24:        r←r−v;25:    **end for**26:    **return** k;27:
**end function**



Combining together the function keySearch with techniques for searching over partitions independently, the authors of the research paper [29] introduced a key search algorithm, described in Algorithm 20. The function KS works by partitioning the search space into sections of which the size follows a geometrically increasing sequence using a size parameter a=O(1). This parameter is chosen such that the number of loop iterations is balanced with the number of keys verified per block.

**Algorithm 19** enumerates and tests key candidates with weight between $W_{1}$ and $W_{2}.$

1:**function**keySearch($W_{1},W_{2},T$)2:    $b\leftarrow \mathrm{initialise}(W_{1},W_{2})$;3:    r←1;4:    **while** True **do**5:        $k\leftarrow \mathrm{getKey}(b,W_{1},W_{2},r)$;6:        **if** k=⊥ **then**7:           **break**;8:        **end if**9:        **if** T(k)=1 **then**10:           **break**;11:        **end if**12:        r←r+1;13:    **end while**14:    **return** k;15:
**end function**



**Algorithm 20** searches key candidates in a range with a size of *e* approximately.
1:**function**KS(e,T)2:    $W_{1}\leftarrow W_{min}$;3:    $W_{2}\leftarrow W_{min}+1$;4:    step←0;5:    Choose $W_{e}$ such that $\mathrm{rank}(0,W_{e})$ is approx *e*;6:    **while** $W_{1}\leq W_{e}$ **do**7:        $k\leftarrow \mathrm{keySearch}(W_{1},W_{2},T)$;8:        **if** k≠⊥ **then**9:           **return** k;10:        **end if**11:        step←step+1;12:        $W_{1}\leftarrow W_{2}$;13:        Choose $W_{2}$ such that $\mathrm{rank}(W_{1},W_{2})$ is approx $a^{step}$;14:    **end while**15:    **return** ⊥;16:
**end function**



Having introduced the function KS, the authors of the research paper [29] transformed it into a quantum key search algorithm that heavily relies on Grover’s algorithm [32]. This is a quantum algorithm to solve the following problem: Given a black box function which returns 1 on a single input *x* and 0 on all other inputs, find *x*. Note that, if there are *N* possible inputs to the black box function, the classical algorithm uses O(N) queries to the black box function since the correct input might be the very last input tested. However, in a quantum setting, a version of Grover’s algorithm solves the problem using $O(N^{1/2})$ queries, with certainty [32,33]. Algorithm 21 describes the quantum search algorithm, which achieves a quadratic speedup over the classical key search (Algorithm 20) [29]. However, it would require significant quantum memory and a deep quantum circuit, making its practical application in the near future rather unlikely.

**Algorithm 21** performs a quantum search of key candidates in a range with a size of *e* approximately.
1:**function**QKS(e,T)2:    $W_{1}\leftarrow W_{min}$;3:    $W_{2}\leftarrow W_{min}+1$;4:    step←0;5:    Choose $W_{e}$ such that $\mathrm{rank}(0,W_{e})$ is approx *e*;6:    **while** $W_{1}\leq W_{e}$ **do**7:        $b\leftarrow \mathrm{initialise}(W_{1},W_{2})$;8:        $f\left(\cdot \right)\leftarrow T(\mathrm{getKey}\left(b,W_{1},W_{2},\cdot \right))$;9:        Call Grover using f for one or zero marked elements in range $\left[W_{1},W_{2}\right)$;10:        **if** marked element *t* found **then**11:           **return** $\mathrm{getKey}(b,W_{1},W_{2},t)$;12:        **end if**13:        step←step+1;14:        $W_{1}\leftarrow W_{2}$;15:        Choose $W_{2}$ such that $\mathrm{rank}(W_{1},W_{2})$ is approx $a^{step}$;16:    **end while**17:    **return** ⊥;18:
**end function**



## 5. Comparison of Key Enumeration Algorithms

In this section, we will make a comparison of the previously described algorithms. We will show some results regarding their overall performance by computing some measures of interest.

### 5.1. Implementation

All the algorithms discussed in this paper were implemented in Java. This is because the Java platform provides the Java Collections Framework to handle data structures, which reduces programming effort, increases speed of software development and quality, and is reasonably performant. Furthermore, the Java platform also easily supports concurrent programming, providing high-level concurrency application programming interfaces (APIs).

### 5.2. Scenario

In order to make a comparison, we will consider a common scenario in which we will run the key enumeration algorithms to measure their performance. Particularly, we generate a random secret key encoded as a bit string of 128 bits, which is represented as a concatenation of 16 chunks, each on 8 bits.

We use a bit-flipping model, as described in Section 3.2. We particularly set α and β to particular values, namely 0.01 and 0.01, respectively. We then create an original key k (AES key) by picking a random value for each chunk *i*, where 0≤i<16. Once this key k has been generated, its bits will be flipped according to the values α and β to obtain a noisy version of it, r. We then use the procedure described in Section 3.2 to assign a score to each of the 256 possible candidate values for each chunk *i*. Therefore, once this algorithm has ended its execution, there will be 16 lists, each having 256 chunk candidates.

These 16 lists are then given to an auxiliary algorithm that does the following. For 0≤i<16, this algorithm outputs 2*^e^*, with 1≤e≤8 chunk candidates for the chunk *i*, ensuring that the original chunk candidate for this chunk is one of the 2*^e^* chunk candidates. This is, the secret key k is one out of all the $2^{16\cdot e}$ key candidates. Therefore, we finally have access to 16 lists, each having 2*^e^* chunk candidates, on which we run each of the key enumeration algorithms. Additionally, on execution, the key candidates generated by a particular key enumeration algorithm are not “tested” but rather “verified” by comparing them to the known key. Note that this is done only for the sake of testing these algorithms; however, in practice, it may be not possible to have such an auxiliary algorithm and the key candidates have to be tested rather than verified.

### 5.3. Results per Algorithms

In order to measure the key enumeration algorithms’ overall performance, we simply generate multiple random instances of the scenario. Once a random instance has been generated, each key enumeration algorithm is run for a fixed number of key candidates. For each run of any algorithm, some statistics are collected, particularly the elapsed time to enumerate a fixed number of key candidates. This was done on a machine with an Intel Xeon CPU E5-2667 v2 running at 3.30 GHz with 8 cores. The set of simulations are run by setting e to 3. Therefore, each list has a size of 8 chunk candidates.

By running the optimal key enumeration algorithm (OKEA) from Section 4.1, we find the following issues: it is only able to enumerate at most $2^{30}$ key candidates, and its overall performance decreases as the number of key candidates to enumerate increases. In particular, the number of key candidates considered per millisecond per core ranges from 2336 in a $2^{20}$ enumeration through 1224 in a $2^{25}$ enumeration to 582 in a $2^{30}$ key enumeration. The main reason for this is that its memory usage grows rapidly as the number of key candidates to generate increases. Indeed, using terminology from Section 4.1.4, we have $W=128=2^{7},w=8=2^{3},$ so a=3,b=4, so this instance of OKEA creates a tree composed of the root node R, the internal nodes $N_{\lambda }^{i_{d}}$ for $0<\lambda \leq 3,0\leq i_{d}<2^{\lambda }$, and the leaf nodes $L^{i}$ for 0≤i<16.

A chunk candidate is a 2-tuple of the form (score,value), where score is a float and value is an integer array. Both a float variable and an integer variable are stored in 32 bits. Now, at level 4, the value has only one entry; therefore, $B_{4}=32+32=64$. At level 3, the value has 2 entries; therefore, $B_{3}=32+2\left(32\right)=96$. At level 2, the value has 4 entries; therefore, $B_{2}=5\left(32\right)=160$. Finally, at level 1, the value has 8 entries; therefore, $B_{1}=9\left(32\right)=288$. After *N* key candidates have been generated, the number of bits $M_{N}$ used to store chunk candidates by the algorithm will be

$$\begin{array}{cc} M_{n} & =\sum_{\lambda =1}^{3}2^{\lambda }B_{\lambda }+B_{4}\left(\sum_{d=0}^{15}8\right)+\sum_{d=1}^{N}\sum_{\lambda =1}^{3}p_{\lambda }^{\left(d\right)}B_{\lambda }\\ \; & =9664+\sum_{d=1}^{N}\left(288\cdot p_{1}^{\left(d\right)}+160\cdot p_{2}^{\left(d\right)}+96\cdot p_{3}^{\left(d\right)}\right) \end{array}$$

Since $1\leq p_{\lambda }^{\left(d\right)}\leq 2^{\lambda }$, for 1≤λ≤3,1≤d≤N, then

$$9664+544\cdot N\leq 9664+\sum_{d=1}^{N}\left(288\cdot p_{1}^{\left(d\right)}+160\cdot p_{2}^{\left(d\right)}+96\cdot p_{3}^{\left(d\right)}\right)\leq 9664+1984\cdot N$$

We also need to include the number of bits used to store extended candidates internally in each priority queue $N_{\lambda }^{i_{d}}.Q$ for $0<\lambda \leq 3,0\leq i_{d}<2^{\lambda }$ and the priority queue R.Q. Therefore, we conclude that, despite all the efforts made for implementing this algorithm in an ingenious way, the algorithm’s scalability is mostly affected by its inherent design rather than by a particular implementation.

On the other hand, the bounded-space key enumeration algorithm (BSKEA) with ω=4, described in Section 4.2, is able to enumerate $2^{30},2^{33},2^{36}$ key candidates. However, it has a dramatic decrease in its overall performance as the number of key candidates to enumerate increases, similar to OKEA’s behaviour. In particular, it is able to enumerate about 4800 key candidates per millisecond per core on average in a $2^{30}$ enumeration, but this value drops to about 1820 key candidates on average in a $2^{36}$ enumeration. The possible reasons for this behaviour are its intrinsic design, its memory consumption, and its implementation. The variant of the bounded-space key enumeration algorithm, introduced in Section 4.4.2, has the same problem as OKEA, i.e., its overall performance (hence, its scalability) is degraded by its excessive memory consumption and it is only able to enumerate up to $2^{30}$ key candidates.

**Figure 5:**
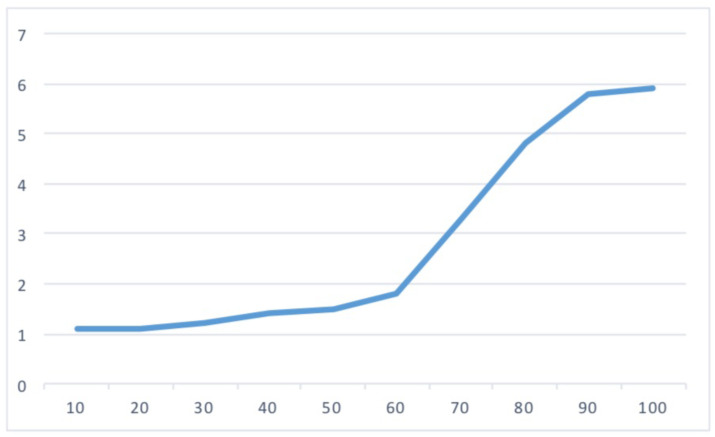
Variation of run times for Algorithm 12 of KEA with histograms from Section 4.8. The *y*-axis represents the run time (milliseconds), while the *x*-axis represents $N_{b}$.

Regarding the key enumeration algorithm using histograms from Section 4.8, we first analyse the algorithm computing the histograms, i.e., Algorithm 12, and the algorithm computing $x_{start},x_{stop}$. These two algorithms were run for $N_{b}=10,20,\ldots ,100$, $R_{1}=1$ and $R_{2}=2^{30}$ for 100 times. We notice that the run time increases as $N_{b}$ increases, especially for Algorithm 12, as Figure 5 shows. On the other hand, the other algorithm shows some negligible variations in its run time. Moreover, as expected, we note that the parameter $N_{b}$ makes the number of bins of $H_{0:N-1}$ increase; therefore, setting this parameter to a proper value helps in guaranteeing the number of key candidates to enumerate, while running through the enumeration bounds $x_{start},x_{stop}$ will be closer to $R_{2}-R_{1}+1=2^{30}=1,073,741,824$. Table 2 shows the number of bins of $H_{0:N-1}$ and the total number of key candidates to be enumerated between bounds $x_{start},x_{stop}$ on average.

Concerning the memory consumed by the arrays used to store histograms, we know that the total number of bits to store all lists $L_{H_{i}}$, 0≤i<16 is given by Equation (1) from Section 4.8.4. Therefore, we set $B_{p}$, which is the number of bits to store a pointer, to 32 bits and set $B_{c}$, the number of bits to store a chunk candidate (score,value), to 64. Therefore, $N\cdot B_{p}\cdot N_{b}+B_{c}\cdot \sum_{i=0}^{N-1}=512\cdot N_{b}+8192.$ Now, the number of bits for storing all the convoluted histograms is given by Equation (2) from Section 4.8.4. We set $B_{int}=32$; therefore, $32\cdot \left(N_{b}-1\right)\frac{\left(15)(16\right)}{2}+\left(32\cdot 15\right)\cdot N_{b}=3840\cdot \left(N_{b}-1\right)+480\cdot N_{b}.$ Table 3 shows the number of bits for storing both standard histograms and convoluted histograms for values $N_{b}=10,30,50,70,$ and 100.

We now report results concerning the enumeration algorithm of KEA with histograms, i.e., Algorithm 14. To run this algorithm, we first set the parameter $R_{1}$ to 1, $R_{2}$ to $2^{z}$, where z=30,33,36, and $N_{b}$ to 60. Once the pre-computation algorithms have ended their execution, we run Algorithm 14 for each index bin in the range calculated by Algorithm 13. Therefore, we find that this algorithm is able enumerate $2^{30},2^{33},2^{36}$ key candidates and that its enumeration rate is between 3500 and 3800 key candidates per millisecond per core. Additionally, as seen, its memory consumption is low.

Concerning the stack-based key enumeration algorithm from Section 4.5, we first compute suitable values for $B_{1}$ and $B_{2}$ by employing the convoluted histogram $H_{0:N-1}$ generated by Algorithm 12. We then run Algorithm 8 with parameters $B_{1}$ and $B_{2}$ but limit the enumeration over this interval to not exceed the number of key candidates to enumerate; this number is obtained from the previous enumeration. Therefore, we find that this algorithm is able to enumerate $2^{30},2^{33},2^{36}$ key candidates and that its enumeration rate is between 3300 and 3500 key candidates per millisecond per core.

Regarding its memory consumption, the stack-based key enumeration algorithm only uses two precomputed arrays, minArray and maxArray, both of which have N+1=17 double entries. Additionally, as pointed out in Section 4.5.3, at any stage of the algorithm, there are at most 16 4-tuples stored in the stack S. Note that a 4-tuple consists of a double entry, two int entries, and an entry holding an int array indices. This array, indices, may have at most 16 entries, each holding an integer value. Therefore, its memory consumption is low.

Lastly, concerning the score-based key enumeration algorithm from Section 4.7, we first run its pre-computation algorithms, i.e., the algorithms for computing the tables minArray, maxArray, and iRange. As was pointed out in Section 4.7.4, the size of table iRange, hence the run time for calculating it, depends heavily on the scaling technique used to get a positive integer (weight) from a real number (score). We particularly use $score\cdot 10^{s}$ with s=4 to get an integer score (weight) from a real-valued score. We find that the table iRange has around 15,066 entries on average. Each of these entries point to a list of integers of which the number of entries is about 4 on average. Therefore, we have that the number of bits to store this table is 64+(32·5)(15,066)=2,410,624 on average. Furthermore, we run Algorithm 11 but limit it to not exceed the number of key candidates to enumerate. As a result, we find that this algorithm can enumerate between 2600 and 3000 key candidates per millisecond per core.

### 5.4. Discussion

From the results discussed in Section 5, it can be seen that all key enumeration algorithms except for the optimal key enumeration algorithm (OKEA) and the variant of BSKEA have a much better overall performance and are able to enumerate a higher number of key candidates. In particular, we find that all of them are able to enumerate $2^{30},2^{33},2^{36}$ key candidates, while OKEA and the variant of BSKEA are only able to enumerate up to $2^{30}$. Their poor performance is caused by their excessive consumption of memory. In particular, OKEA is the most memory-consuming algorithm, hence degrading its overall performance and scalability. In general, scalability is low in optimal key enumeration algorithms [18,28], considering that not too many candidates can be enumerated as a result of the exponential growth in their memory consumption. However, by relaxing the restriction on the order in which the key candidates will be enumerated, we are able to design nonoptimal key enumeration algorithms, having better overall performance and scalability. In particular, relaxing this restriction on the order allows for the construction of parallelizable and memory-efficient key enumeration algorithms, as was evinced in this paper and the results previously described. Moreover, all the algorithms save for OKEA [12,13,14,15,16,17] as described in this paper are nonoptimal ones, and their respective descriptions and empirical results show that they are expected to have a better overall performance and to consume much less computational resources. Table 4 briefly summarises some qualitative and functional attributes of the described algorithms.

Additionally, note that, when an array is used to store a private key and each entry of this array contains much more data than required in the sense that the number of bits used to store a reduced set of numbers is greater than required, this redundancy as well as the small number of candidates per chunk allow us to generate more “reliable” scores for the candidates per chunk (which would make the key enumeration algorithms find the correct key after enumerating much fewer candidates). From an implementer’s view, this may be mitigated by reducing the redundancy used to store a particular private key.

## 6. Conclusions

In this paper, we investigated the key enumeration problem, since there is a connection between the key enumeration problem and the key recovery problem. The key enumeration problem arises in the side-channel attack literature, where, for example, the attacker might procure scoring information for each byte of an AES key from a power analysis attack [34] and then want to efficiently enumerate and test a large number of complete 16-byte candidates until the correct key is found.

In summary, we first stated the key enumeration problem in a general way and then studied and analysed several algorithms to solve this problem, such as the optimal key enumeration algorithm (OKEA); the bounded-space near-optimal key enumeration algorithm; the simple stack-based, depth-first key enumeration algorithm; the score-based key enumeration algorithm; and the key enumeration algorithm using histograms. For each studied algorithm, we described its inner functioning, showing its functional and qualitative features, such as memory consumption, amenability to parallelization, and scalability. Furthermore, we proposed variants of some of them and implemented all of them on Java. We then experimented with them and made an experimental comparison of all of them, drawing special attention to their strengths and weaknesses.

As a future research, it would be interesting to find cryptanalysis scenarios to which we could apply key enumeration algorithms together with other techniques. For example, we can think of evaluating the post-quantum cryptographic schemes submitted to the second round of the National Institute of Standards and Technology (NIST) post-quantum cryptography standardization process in the cold boot attack setting [10]. Furthermore, we can think of exploring the use of key enumeration algorithms in cache attacks to achieve full key recovery when insufficient information is gathered [35].

## Figures and Tables

**Table 1:** Brief description of reviewed key enumeration algorithms (KEAs).

	Properties	
Algorithm Name	Is it order optimal?	Is it customizable?
Optimal KEA	Yes	No, inherently serial
Bounded-Space Near-Optimal KEA	Near-optimal if not parallelized	No
Stack-Based KEA	No	Yes, parametrized by a given interval
Threshold	No	Yes, parametrized by a given interval
Weight-Based KEA	Near-optimal if properly parametrized	Yes, parametrized by a given interval
KEA with Histograms	No	Yes, parametrized by a given interval
Quantum KEA	No	Yes, parametrized by a given interval

**Table 2:** Variation of the total number of key candidates to be enumerated between bounds $x_{start},x_{stop}$ on average.

Value for $N_{b}$	Size of List *L*_*H*0:_${}_{N}$_−1_	Total Count of Key Candidates
10	145	1,412,497,166
20	224	1,310,161,019
30	305	1,260,927,932
40	384	1,228,979,005
50	464	1,207,956,426
60	545	1,191,780,722
70	625	1,178,891,769
80	705	1,169,493,889
90	784	1,162,092,971
100	864	1,156,185,368

**Table 3:** Memory consumption in bits for storing histograms.

Values for *N_b_*	Bit Count for Standard Histograms	Bit Count for Convoluted Histograms	Total Bit Count
10	13,312	39,360	52,672
30	23,552	125,760	149,312
50	33,792	212,160	245,952
70	44,032	298,560	342,592
100	59,392	428,160	487,552

**Table 4:** Qualitative and functional attributes of key enumeration algorithms.

	Properties		
Algorithm Name	Parallelizable	Memory Consumption	Scalability
Optimal KEA	No	High	Low
Bounded-Space
Near-Optimal KEA	Yes, but loses its near-optimality property	Moderate	Moderate
Stack-Based KEA	Yes	Low	High
Threshold	Yes, but only works for a first task	Depends how L is stored	High
Weight-Based KEA	Yes	Low	High
KEA with Histograms	Yes	Low	High
Quantum KEA	Yes	High	High

## References

[1] Halderman J.A., Schoen S.D., Heninger N., Clarkson W., Paul W., Calandrino J.A., Feldman A.J., Appelbaum J., Felten E.W. Lest We Remember: Cold Boot Attacks on Encryption Keys. Proceedings of the 17th USENIX Security Symposium.

[2] Heninger N., Shacham H. Reconstructing RSA Private Keys from Random Key Bits. Proceedings of the 29th Annual International Cryptology Conference.

[3] Henecka W., May A., Meurer A. Correcting Errors in RSA Private Keys. Proceedings of the 30th Annual Conference on Advances in Cryptology.

[4] Paterson K.G., Polychroniadou A., Sibborn D.L. A Coding-Theoretic Approach to Recovering Noisy RSA Keys. Proceedings of the 18th International Conference on the Theory and Application of Cryptology and Information Security.

[5] Lee H.T., Kim H., Baek Y.J., Cheon J.H. Correcting Errors in Private Keys Obtained from Cold Boot Attacks. Proceedings of the 14th International Conference on Information Security and Cryptology.

[6] Poettering B., Sibborn D.L. Cold Boot Attacks in the Discrete Logarithm Setting. Proceedings of the Cryptographer’s Track at the RSA Conference 2015.

[7] Albrecht M., Cid C. Cold Boot Key Recovery by Solving Polynomial Systems with Noise. Proceedings of the 9th International Conference, ACNS 2011.

[8] Kamal A.A., Youssef A.M. Applications of SAT Solvers to AES Key Recovery from Decayed Key Schedule mages. Proceedings of the 2010 Fourth International Conference on Emerging Security Information, Systems and Technologies.

[9] Albrecht M.R., Deo A., Paterson K.G. (2018). Cold Boot Attacks on Ring and Module LWE Keys Under the NTT. IACR Trans. Cryptogr. Hardw. Embed. Syst..

[10] Paterson K.G., Villanueva-Polanco R. Cold Boot Attacks on NTRU. Proceedings of the 18th International Conference on Cryptology in India.

[11] Villanueva-Polanco R. Cold Boot Attacks on Bliss. Proceedings of the 6th International Conference on Cryptology and Information Security in Latin America.

[12] Bogdanov A., Kizhvatov I., Manzoor K., Tischhauser E., Witteman M. Fast and Memory-Efficient Key Recovery in Side-Channel Attacks. Proceedings of the 22nd International Conference.

[13] David L., Wool A. A Bounded-Space Near-Optimal Key Enumeration Algorithm for Multi-subkey Side-Channel Attacks. Proceedings of the Cryptographers’ Track at the RSA Conference.

[14] Longo J., Martin D.P., Mather L., Oswald E., Sach B., Stam M. (2016). How Low Can You Go? Using Side-Channel Data to Enhance Brute-Force Key Recovery. http://eprint.iacr.org/2016/609.

[15] Martin D.P., Mather L., Oswald E., Stam M. Characterisation and Estimation of the Key Rank Distribution in the Context of Side Channel Evaluations. Proceedings of the International Conference on the Theory and Application of Cryptology and Information Security.

[16] Martin D.P., O’Connell J.F., Oswald E., Stam M. Counting Keys in Parallel After a Side Channel Attack. Proceedings of the International Conference on the Theory and Application of Cryptology and Information Security.

[17] Poussier R., Standaert F.X., Grosso V. Simple Key Enumeration (and Rank Estimation) Using Histograms: An Integrated Approach. Proceedings of the 18th International Conference.

[18] Veyrat-Charvillon N., Gérard B., Renauld M., Standaert F.X. An Optimal Key Enumeration Algorithm and Its Application to Side-Channel Attacks. Proceedings of the International Conference on Selected Areas in Cryptography.

[19] Veyrat-Charvillon N., Gérard B., Standaert F.X. Security Evaluations beyond Computing Power. Proceedings of the Annual International Conference on the Theory and Applications of Cryptographic Techniques.

[20] Bernstein D.J., Lange T., van Vredendaal C. (2015). Tighter, Faster, Simpler Side-Channel Security Evaluations beyond Computing Power. http://eprint.iacr.org/2015/221.

[21] Ye X., Eisenbarth T., Martin W., Joye M., Moradi A. (2015). Bounded, yet Sufficient? How to Determine Whether Limited Side Channel Information Enables Key Recovery. Smart Card Research and Advanced Applications.

[22] Choudary M.O., Popescu P.G. Back to Massey: Impressively Fast, Scalable and Tight Security Evaluation Tools. Proceedings of the International Conference on Cryptographic Hardware and Embedded Systems.

[23] Choudary M.O., Poussier R., Standaert F.X. Score-Based vs. Probability-Based Enumeration—A Cautionary Note. Proceedings of the International Conference on Cryptology in India.

[24] Glowacz C., Grosso V., Poussier R., Schüth J., Standaert F.X. Simpler and More Efficient Rank Estimation for Side-Channel Security Assessment. Proceedings of the International Conference on Fast Software Encryption.

[25] Poussier R., Grosso V., Standaert F.X., Homma N., Medwed M. (2016). Comparing Approaches to Rank Estimation for Side-Channel Security Evaluations. Smart Card Research and Advanced Applications.

[26] Grosso V., Bilgin B., Fischer J.B. (2019). Scalable Key Rank Estimation (and Key Enumeration) Algorithm for Large Keys. Smart Card Research and Advanced Applications.

[27] Junod P., Vaudenay S., Johansson T. (2003). Optimal Key Ranking Procedures in a Statistical Cryptanalysis. Fast Software Encryption.

[28] Seshadri N., Sundberg C.W. (1994). List Viterbi decoding algorithms with applications. IEEE Trans. Commun..

[29] Martin D.P., Montanaro A., Oswald E., Shepherd D.J. Quantum Key Search with Side Channel Advice. Proceedings of the International Conference on Selected Areas in Cryptography.

[30] Cormen T.H., Leiserson C.E., Rivest R.L., Stein C. (2009). Introduction to Algorithms.

[31] Martin D.P., Mather L., Oswald E. Two Sides of the Same Coin: Counting and Enumerating Keys Post Side-Channel Attacks Revisited. Proceedings of the Cryptographers’ Track at the RSA Conference.

[32] Grover L.K. (1996). A Fast Quantum Mechanical Algorithm for Database Search. Proceedings of the Twenty-eighth Annual ACM Symposium on Theory of Computing.

[33] Grover L.K. (1997). Quantum Mechanics Helps in Searching for a Needle in a Haystack. Phys. Rev. Lett..

[34] Mangard S., Oswald E., Popp T. (2007). Power Analysis Attacks: Revealing the Secrets of Smart Cards (Advances in Information Security).

[35] Yarom Y., Genkin D., Heninger N. CacheBleed: A Timing Attack on OpenSSL Constant Time RSA. Proceedings of the Workshop on Cryptographic Hardware and Embedded Systems.

